# IL-2 and IL-15 drive intrathymic development of distinct periphery-seeding CD4^+^Foxp3^+^ regulatory T lymphocytes

**DOI:** 10.3389/fimmu.2022.965303

**Published:** 2022-09-08

**Authors:** Cécile Apert, Ariel O. Galindo-Albarrán, Sarah Castan, Claire Detraves, Héloise Michaud, Nicola McJannett, Bart Haegeman, Simon Fillatreau, Bernard Malissen, Georg Holländer, Saulius Žuklys, Jérémy C. Santamaria, Olivier P. Joffre, Paola Romagnoli, Joost P. M. van Meerwijk

**Affiliations:** ^1^ Toulouse Institute for Infectious and Inflammatory Diseases (Infinity), INSERM UMR1291 – CNRS UMR5051 – University Toulouse III, Toulouse, France; ^2^ Station d’Ecologie Théorique et Expérimentale, CNRS, Moulis, France; ^3^ Institut Necker Enfants Malades, Inserm U1151, CNRS UMR8253, Paris, France; ^4^ Université de Paris Descartes, Faculté de Médecine, Paris, France; ^5^ AP-HP, Hôpital Necker-Enfants Malades, Paris, France; ^6^ Centre d’Immunophénomique (CIPHE), Aix Marseille Université, INSERM, CNRS, Marseille, France; ^7^ Paediatric Immunology, Department of Biomedicine, University of Basel and University Children’s Hospital Basel, Basel, Switzerland; ^8^ Department of Paediatrics and the Weatherall Institute of Molecular Medicine, University of Oxford, Oxford, United Kingdom; ^9^ Department of Biosystems Science and Engineering, ETH Zurich, Basel, Switzerland

**Keywords:** thymus, T lymphocyte, regulatory T cell (T reg), immunopathology, cytokines

## Abstract

Development of Foxp3-expressing regulatory T-lymphocytes (Treg) in the thymus is controlled by signals delivered in T-cell precursors *via* the TCR, co-stimulatory receptors, and cytokine receptors. In absence of IL-2, IL-15 or their receptors, fewer Treg apparently develop in the thymus. However, it was recently shown that a substantial part of thymic Treg are cells that had recirculated from the periphery back to the thymus, troubling interpretation of these results. We therefore reassessed the involvement of IL-2 and IL-15 in the development of Treg, taking into account Treg-recirculation. At the age of three weeks, when in wt and IL-15-deficient (but not in IL-2-deficient) mice substantial amounts of recirculating Treg are present in the thymus, we found similarly reduced proportions of newly developed Treg in absence of IL-2 or IL-15, and in absence of both cytokines even less Treg developed. In neonates, when practically no recirculating Treg were found in the thymus, the absence of IL-2 led to substantially more reduced Treg-development than deficiency in IL-15. IL-2 but not IL-15 modulated the CD25, GITR, OX40, and CD73-phenotypes of the thymus-egress-competent and periphery-seeding Treg-population. Interestingly, IL-2 and IL-15 also modulated the TCR-repertoire expressed by developing Treg. Upon transfer into Treg-less *Foxp3^sf^* mice, newly developed Treg from IL-2- (and to a much lesser extent IL-15-) deficient mice suppressed immunopathology less efficiently than wt Treg. Taken together, our results firmly establish important non-redundant quantitative and qualitative roles for IL-2 and, to a lesser extent, IL-15 in intrathymic Treg-development.

## Introduction

Regulatory T lymphocytes expressing the forkhead/winged helix transcription factor Foxp3 (Treg) play a central role in the control of innate and adaptive immune responses (1). This is best illustrated by the observation that the absence of Treg in individuals and animals carrying mutations in the gene encoding Foxp3 leads to a rapidly lethal inflammatory autoimmune syndrome (2, 3). The thymus is the major organ where Treg development occurs, even if Treg can also differentiate from conventional T cells (Tconv) in the periphery (4). In the thymus, development of T cell precursors into either Treg or Tconv is governed by several parameters including signals transmitted by the TCR, co-stimulatory receptors, and cytokine receptors. Thus, it was shown that high affinity interactions between the precursor’s TCR and MHC/peptide complexes expressed by thymic stromal cells are required for Treg development (5–7), which results in a Treg population enriched in autospecific cells (as compared to the Tconv-population) (8, 9). Several co-stimulatory molecules are selectively implicated in the development of Treg, including CD28, LFA-1, and CD27 (10–13). Finally, also cytokines appear important for Treg development (14).

Previous reports indicated roles for the cytokines IL-2 and IL-15 in the intrathymic development of Treg (15–23). Mice deficient in IL-2, IL-15, the IL-2-receptor α chain CD25, the β chain shared between the receptors for IL-2 and IL-15 (CD122), or the “common” cytokine-receptor γ chain (γ_c_, CD132), all have reduced proportions of Treg in the thymus. *In vitro*, these cytokines drive differentiation of CD25^+^Foxp3^−^ Treg precursors to fully mature Treg (21). IL-2 was reported to prevent apoptotic cell death of autospecific Treg-precursors and appears to induce Foxp3-expression (22, 24). IL-15 was shown to be involved in the development of CD25^−^Foxp3^+^ (but not CD25^+^Foxp3^−^) Treg precursors (19). Similar mechanisms apparently also operate in the human thymus (25, 26). However, in at least one report unaltered numbers of TCR-transgenic Treg developed in absence of IL-2 (27). Moreover, a large fraction of thymic Treg are cells that had recirculated from the periphery back to the thymus (28). Since IL-2 is required for peripheral survival of Treg (29), this recirculation-process would be expected to be strongly reduced in mice deficient in IL-2, which would, at least in part, explain reduced Treg levels in mice carrying a null-mutation of the *Il2* locus (*Il2°*). Therefore, the roles of IL-2 and IL-15 in development of Treg remain unclear.

In the thymus, IL-2 and IL-15 appear to be produced by stromal cell-types involved in selection of the TCR-repertoire expressed by Treg, including dendritic cells (DC) and medullary epithelial cells (mTEC), as well as by T lymphocytes, but this issue remains controversial (19, 30–34). Through trans-presentation by their respective high affinity receptor α-chains, IL-2 and IL-15 can have very local effects (19, 31, 35). Distinct thymic stromal cell-types have distinct phenotypes and thus apparently convey distinct signals to developing T cells (36). It is therefore conceivable that Treg developing in an IL-2 vs. IL-15-dependent manner are phenotypically and/or functionally distinct.

Mice deficient in IL-2 or its receptor develop a severe and rapidly lethal autoimmune pathology (37–40), which is at least in large part due to the requirement of this cytokine for peripheral survival and function of Treg (29). Also mice deficient in IL-15 or its trans-presenting receptor α-chain develop symptoms of autoimmune-disease, though much later and much less severely. Defects in thymic negative selection of autospecific precursors of CD4^+^ T cells may be involved in the development of these symptoms (32). It remains to be investigated if IL-2 and IL-15 modulate the selection of the TCR-repertoire expressed by Treg developing in the thymus and/or their functional potential, both of which may be involved in the development of symptoms of autoimmune-pathology.

We reassessed the involvement of IL-2 and IL-15 in Treg-development using mice in which we could distinguish newly developed from recirculating Treg. Thus, we firmly confirm that in IL-2- and, to a lesser extent, in IL-15-deficient mice substantially less Treg develop in the thymus. Our data also reveal that these cytokines drive development of thymus-egress and periphery-seeding competent Treg that are phenotypically distinct and that express partly distinct TCR-repertoires. Upon adoptive transfer into new-born Treg-deficient mice, thymus-exit-competent Treg that had developed in IL-2-deficient mice protected less efficiently from immune-pathology than Treg from wt or IL-15-deficient mice. Finally, we found that whereas the IL-2 involved in Treg development is non-redundantly produced by T cells and by DC, the IL-15 appears, at least in part, derived from DC. Based on our and previously reported data, we discuss mechanisms potentially involved in our finding that IL-2 and IL-15 apparently drive development of different Tregs.

## Materials and methods

### Mice

*Rag2-Gfp* mice (41, 42) were kindly provided by Drs. A. Liston and P. Fink; *Foxp3-Thy1^a^* mice (43) by Dr. A. Liston; *Il15°* mice (44) by Dr. Y. Tanriver; and CD4-Cre (45) and CD11c-Cre mice (46) by Dr. J.-C. Guéry. Kaa *Tcrb*-transgenic mice (7) and β5t-Cre mice (47) were previously described. *Il2°* (B6.129P2-*Il2^tm1Hor^*/J) mice, *Tcra°* mice, and *Foxp3^sf^* mice were purchased from JAX laboratories, *Il15^fl^* (C57BL/6N-*Il15^tm1c(EUCOMM)Hmgu^*/H) mice (48) from MRC Harwell Institute/Mary Lyon Centre, Oxfordshire, UK. *Il2^fl^* mice were generated as described in the **Supplementary Materials** and Methods section. All animals were on a C57BL/6 genetic background.

Rare sick animals, identified based on abnormally high proportions of CD4SP cells (>10% of total thymocytes), were excluded from analysis. *Il2^fl^* or *Il15^fl^* mice with germline recombination (as determined by PCR on tail biopsies) were excluded from analysis. Because mice were analysed before sexual maturity, we have not observed any differences between male and female mice, and all data are pools of both sexes.

### Generation of *Il2^flox^* knock-in mice

The mouse *Il2* gene (ENSMUSG00000027720) was edited using a double-stranded homology-directed repair (HDR) template (targeting vector) with 3.5 and 3.3 kb-long 5’ and 3’ homology arms, respectively. It included a first loxP site located 173 bp upstream of exon 3, a second loxP site located 150 bp downstream of exon 3, and a frt-neo^r^-frt cassette. The final targeting vector was abutted to a cassette coding for the diphtheria toxin fragment A (49). JM8.F6 C57BL/6N ES cells (50) were electroporated with 20 mg of targeting vector. After selection in G418, ES cell clones were screened for proper homologous recombination by Southern blot and PCR analysis. A neomycin specific probe was used to ensure that adventitious non-homologous recombination events had not occurred in the selected ES clones. Mutant ES cells were injected into BalbC/N blastocysts. Following germline transmission, excision of the frt-neo^r^-frt cassette was achieved through genetic cross with transgenic mice expressing a FLP recombinase under the control of the actin promoter (51). A pair of primers (sense 5’-GCCACAGAATTGAAAGATCTTC-3’ and antisense 5’-TCTTGTGGAATTCTACTCCG-3’) amplified a 418 bp-long band in the case of the wild-type *Il2* allele and a 500 bp-long band in the case of the mutant LoxP-flanked *Il2* ^flox^ allele.

The resulting *Il2* ^flox^ knock-in mice (official name B6-*Il2^Tm1Ciphe^* mice**)** have been established on a C57BL/6N background. When bred to mice that express tissue-specific Cre recombinase, the resulting offspring will have exon 3 removed in Cre-expressing cells, preventing them to produce IL-2. Germline-recombination was tested by tail DNA genotyping using 5’-AGATTGGAACAATAGTCTGAACTTGTGCT-3’, 5’-TTGCAGGTGATGGTAGGTGGAAAT-3’, and 5’-TCAAATCCAGAACATGCCGCA-3’ primers, allowing to detect 633bp, 755bp, and 245bp bands corresponding to wt, *Il2* ^flox^ and recombined *Il2* ^flox^ alleles, respectively. Requests for *Il2* ^flox^ mice should be addressed to BM.

### Flow cytometry

Sample preparation and staining were performed using standard procedures. S1P1 staining was performed on ice as follows: after blocking non-specific labelling with mouse IgG (100μg/ml), cells were stained with unlabelled anti-S1P1 (50μg/ml, 90’), then stained with donkey-anti-rat IgG-biotin (1/100 diluted) in presence of mouse IgG (100 μg/ml, 30’), then blocked with rat anti-mouse FcγR antibody 2.4G2 (10 μg/ml, 15’) and then incubated with streptavidin-PE (30’). Finally, staining with antibodies to indicated surface markers was performed in presence of 2.4G2 (10 μg/ml, 30’). Antibodies are listed in **Table S1A**. For MOG (35–55)/I-A^b^ tetramer (NIH tetramer facility) staining, after organ digestion, cells were washed, resuspended in RPMI medium, and incubated 15min on ice with an Fc block mix (2.4G2 at 10μg/ml; mouse and rat IgG at 25μg/ml) and 100nM dasatinib (Sigma-Aldrich). 6x10^6^ cells were then incubated for 2 hours at 25°C with 1.5 μl tetramer in 100 μl RPMI medium. Labelled cells were acquired using an LSRII or a Fortessa flow cytometer (BD Biosciences, San Jose, CA) and the data analysed using FlowJo software (Tree Star, Ashland, OR). Doublets and dead cells were excluded from the analysis by using appropriate FSC/SSC gates.

### Generation of TCRseq libraries

CD4^+^CD8^−^Thy1.1^+^GFP^+^ thymic Treg (105) were FACS sorted from individual three-week-old wt (n=4), *Il2*° (n=4), or *Il15*° (n=4) *Rag2-Gfp Foxp3-Thy1a Tcra^+/o^* Kaa TCRβ-transgenic B6 mice. RNA was extracted by Nucleospin RNA XS (Macherey-Nagel) according to the manufacturer´s instructions, and was quality controlled (RIN > 8) using Agilent 2100 BioAnalyzer (Agilent technology). cDNA synthesis and library preparation were performed as previously described (52), and was adjusted to our different conditions. In brief, cDNA synthesis was performed in a thermocycler using 1 μM of reverse transcription oligonucleotides mixture corresponding to the TCRα constant region (**Table S1B**: TRAC_RT_1-9), a DNA-RNA hybrid template-switch oligonucleotide with 12 random nucleotides serving as unique molecular identifier (UMI) to tag individual mRNA molecules (**Table S1B**: UNIV5_TSv2), 5 U/μl of SMART Scribe reverse transcriptase (Clontech), 2 U/μl of Recombinant RNase inhibitor (Clontech), 0.5 mM of each dNTP, Ultra low fast first strand buffer, 5mM of DTT, 1M of Betaine, 6 mM of MgCl_2_, incubated during 45’ at 42°C, 10’ at 70°C. After removal of hybrid oligonucleotide with 1U of Uracil-DNA Glycosylase (Biolabs) incubated during 40’ at 37°C, the cDNA was purified using Agenecourt AMPure XP beads (Beckman Coulter) according to the manufacturer´s instructions. The first PCR reaction was performed with 0.2 μM of the oligonucleotides UNIV5_P12v2 and TRAC3_P1v2 (**Table S1B**), in the PCR-mix-solution (manufacturer’s buffer with 1.5 mM MgSO_4_, 0.2 mM of each dNTP, and 0.02 U/μL Hot Start DNA Polymerase (Millipore)), with the parameters 2’ at 95°C; 10 cycles of 20” at 95°C, 15” at 59°C, 45” at 70°C; and a final incubation of 3.5’ at 70°C. The amplicons were then purified using Agenecourt AMPure XP beads. The second, semi-nested PCR was done using 2’ at 95°C followed by 20 cycles of 20” at 95°C, 15” at 59°C, 45” at 70°C; and a final incubation of 3.5’ at 70°C with UNIV5_P12v2 and TRAC3_P2v2 (**Table S1B**). In the third PCR a 3’ index, P5 and P7 Illumina sequences, and read1, read2, and index sequencing sequences were added. It was performed using UNIV5_P3v2 and TRAC3_P3v2-index (**Table S1B**), as follows: 2’ at 95°C, 1 cycle of 20” at 95°C, 15” at 59°C, 45” at 70°C; 5 cycles of 20” at 95°C, 15” at 75°C, 45” at 70°C; and a final incubation of 3.5’ at 70°C. For the fourth amplification PCR, primers UNIV5_P4v2 and UNIV3_P4v2 (**Table S1B**) and thermocycler parameters 2’ at 95°C, 5 cycles of 20” at 95°C, 15” at 60°C, 45” at 70°C; and a final incubation of 3.5’ at 70°C, were used. The quality of each library was checked by using Agilent 2100 BioAnalyzer with a 640pb mean peak size. The samples were indexed and sequenced with 300pb paired end on Illumina MiSeq sequencer (Illumina).

### Processing of TCRseq data

Initially, reads were processed with the toolkit pRESTO (53) as follows. Using FilterSeq, reads with a quality higher than 20 were selected. Using MaskPrimers and PairSeq algorithms, the sequences corresponding to the *Tcra* constant region (AGCAGGTTCTGGGTTCTGGA) and indicating location of the UMI (CTTGGGGG) were searched for and indexed to the head of the paired reads. Using BuildConsensus, consensus-sequences of the reads with the same UMI were constructed. Next, the forward and reverse reads were aligned to assemble the *Tcra* sequences (AssemblePairs) and the UMI groups containing at least two reads were selected. The sequenced fragments from each selected UMI were aligned to the *Tcra* genomic region using the toolkit MiXCR (54), with the tools “align” and “assemble”. The aligned fragments were exported as data tables “clonotype-tables” using the tool “exportClones”. Using VDJtools (55) these clonotype-tables were then processed to graph with customized scripts in R. For all graphics described below, the “clonotypes” were selected according to their differences in the amino-acid sequences of the V segments, CDR3, and J segments. The Chao1 and Shannon-Wiener diversity-measures of the total *Tcra* repertoires were calculated using the command CalcDiversityStats from VDJtools. The Morisita-Horn similarity measure of clonotypes represented at ≥ 5 UMIs in individual samples was determined using the R package “divo”. “Public repertoires” were defined as the clonotypes present in all four replicates for each condition. Custom scripts used are available at https://github.com/arielgalindoalbarran/IL2_IL15_Tregdependents.

### *In vivo* Treg-assays

New-born (*Foxp3^sf^Rag2° x Foxp3^sf^Tcra°*)F1 mice were i.v. injected into the temporal vein (56) with 4x10^5^ GFP^+^CD4^+^CD8^−^Thy1.1^+^ Treg cells FACS-sorted from *Rag2-Gfp Foxp3-Thy1^a^* thymi. At three weeks of age, mice were euthanized, macroscopically analysed, and blood and organs collected for analysis.

### Determination of antibody titres and of autoantibodies

Serum antibody titres were determined using LegendPlex (BioLegend), according to the manufacturer’s instructions. For tissue-immunoblots, organs/tissues were harvested from RAG2-deficient B6 mice, rinsed in PBS, and lysed with a Dounce homogenizer in RIPA buffer containing a protease inhibitor cocktail. The crude tissue extracts were centrifuged (10^4^G, 12 min) and the soluble protein extracts were aliquoted and stored at -80°C. The protein concentration was determined using standard Bradford Protein Assay. 35 μg of total soluble protein-extract was run on SDS-PAGE, then blotted on a nitrocellulose membrane. Membranes were incubated in Odyssey blocking buffer (Li-Cor) for 30’ at RT, then incubated o/n at 4°C with 800-fold-diluted sera from *scurfy* mice injected or not with Treg. Bound immunoglobulin was detected using IRDye^®^ 800CW-labeled Goat-anti-Mouse IgG(1/2a/2b/3)(LiCor, 1h at RT). This antibody also reacts with Igκ and IgΛ. Fluorescence was visualized and quantified using the Odyssey Classic Imaging System and ImageStudio software (Li-Cor).

## Data and materials availability

The TCRseq data reported in this study are available from Gene Expression Omnibus with accession code GSE153484. Mice are available upon request.

## Statistical analysis

The statistical significance of differences between groups of data was analysed using the two-tailed Mann-Whitney test or the Wilcoxon matched pairs signed rank test, as indicated.

## Results

### IL-2 and IL-15 play major non-redundant quantitative roles in intrathymic Treg development

**Figure 1:**
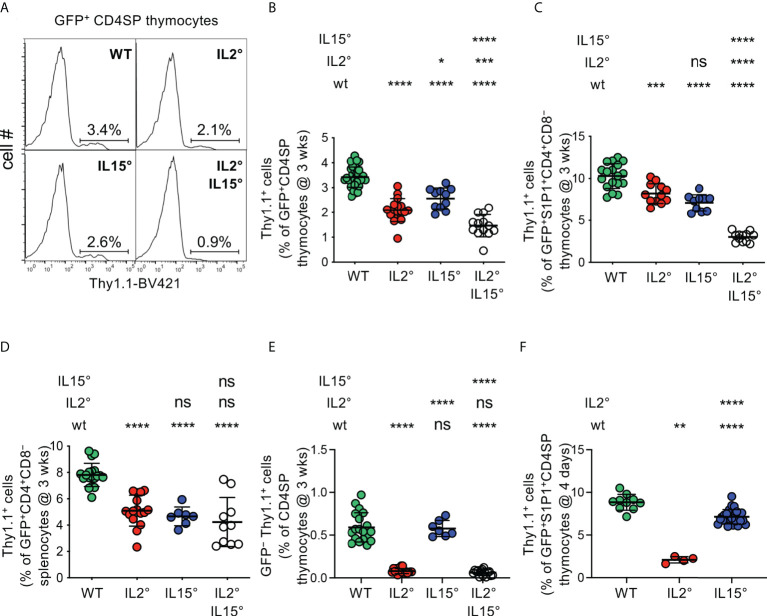
*IL-2 and IL-15 quantitatively regulate intrathymic Treg-development.* Thymocytes from **(A–E)** three-week-old or **(F)** four-day-old *Rag-Gfp Foxp3-Thy1^a^* mutant, *Il2°* and/or *Il15°* mice, and *Il2^wt/wt^* and *Il15^wt/wt^* littermates (for absolute numbers see **Figure S1A**), were stained with fluorescent antibodies against indicated markers and analysed by flow cytometry. **(A)** Representative histograms of Thy1.1 expression (indicating Foxp3-expression) on GFP^+^ (*i.e.* newly developed) CD4^+^CD8^−^TCR^high^ (CD4SP) thymocytes (gated as in **Figure S1B**). Indicated gates were used for the quantification of Thy1.1^+^ Treg. **(B)** Percentages of Treg among GFP^+^ CD4SP thymocytes from indicated mice (n=31, 17, 11, and 13 wt, *Il2°, Il15°*, and *Il2°Il15°* mice, respectively). **(C)** Percentages of Treg among GFP^+^ S1P1^+^ CD4^+^CD8^−^ thymocytes (n=18, 12, 10, and 12 mice). For S1P1 gates see **Figure S1E**. **(D)** Percentages of Treg among RTE in the spleen (n=19, 16, 7, 10 mice). For RTE gates see **Figure S1F**. **(E)** Percentages of GFP^−^ (recirculating) Treg among CD4SP thymocytes (n=19, 17, 7, and 15 mice). For recirculating Treg-gates see **Figure S1G**. **(F)** Percentages of Treg among GFP^+^ S1P1^+^ CD4SP thymocytes from four-day-old mice (n=10, 4, and 25 mice) For absolute numbers see **Figure S1H**. Dots indicate individual mice. ns, not significant; *p < 0.05; **p < 0.01; ***p < 0.001; ****p < 0.0001 (Mann-Whitney test). Bars indicate mean values ± SD.

We and others recently showed that a substantial proportion of the thymic Treg-pool is composed of cells that had recirculated from the periphery back to the thymus (28, 57–60). Thus, in young B6 adults, typically analysed in reports, 40 to 80% of thymic Treg are recirculating cells (28). This issue was not taken into account in the studies demonstrating reduced numbers of Treg in the thymus of mice deficient for IL-2, IL-15, or their receptors, and may dramatically impinge on interpretation of results. We therefore first reassessed the respective roles of IL-2 and IL-15 in intrathymic differentiation of Treg strictly focusing on newly developed Treg. To do so, we used mutant mice expressing green-fluorescent-protein (GFP) under control of the *Rag2* promoter and the allelic cell-surface marker Thy1.1 under control of the *Foxp3* promoter (*Rag2-Gfp Foxp3-Thy1^a^*). In the thymi of such mice, GFP^+^Thy1.1^+^ and GFP^−^Thy1.1^+^ cells are newly developed and recirculating/thymus-resident Treg, respectively (28). Initial analyses were conducted on thymi of three-week-old mice to avoid any potential confounding effects due to the thymic involution resulting from the immune-pathology developing later-on in IL-2-deficient mice. Importantly, Treg developing early in life play a central role in the prevention of lethal immunopathology (61), validating this choice. We compared IL-2- and/or IL-15-deficient (*Il2° and Il15°*, respectively), *Rag2-Gfp Foxp3-Thy1a* mice with *Il2^wt/wt^* or *Il15^wt/wt^* (“wt”) littermates. The absolute numbers of total thymocytes recovered from wt, *Il2°*, and *Il15°* mice were similar (**Figure S1A**). We determined the percentages of Foxp3^+^ Treg among newly developed (GFP^+^) CD4^+^CD8^−^TCR^high^ (CD4SP) thymocytes (**Figures S1B**, **1A**) and thus observed that in the absence of IL-2 substantially (39 ± 14%) less Foxp3^+^ Treg developed in the thymus (**Figure 1B**). Remarkably, a similar analysis revealed that, as compared to wt littermates, also in *Il15°* mice substantially (25 ± 12%) less Treg newly developed. Mice lacking both IL-2 and IL-15 (*Il2°Il15°* mice) displayed the strongest decrease in newly developed Treg among CD4SP cells (reduction of 74 ± 11%). These effects were specific to Treg as the development of CD4SP Tconv was not much affected (**Figure S1C**). Further analysis of the absolute numbers of newly developed Treg in the thymi of wt and mutant mice confirmed that IL-2 and IL-15 play quantitatively non-redundant roles in intrathymic Treg development (**Figure S1D**).

Negative selection through induction of apoptosis can happen up to late stages of T cell development (62). To assess the involvement of IL-2 and IL-15 in Treg development up to late stages of this process, we therefore first quantified developing Treg expressing the sphingosine 1-phosphate receptor (S1P1), sufficient for thymic egress of T lymphocytes (63). As expected, a large proportion of Treg expressed S1P1 (**Figure S1E**). Analyses of thymocytes from wt and cytokine-mutant mice revealed substantially lower levels of Treg among newly developed GFP^+^S1P1^+^CD4^+^CD8^−^ thymocytes in *Il2°* and in *Il15°* mice, as compared to wt littermates (reductions of 20 ± 11% and 31 ± 9%, respectively), with a defect that was most pronounced in *Il2°Il15°* mice (reduction of 70 ± 6%, **Figure 1C**). To assess involvement of IL-2 and IL-15 up to the very last stage of intrathymic Treg development, we also assessed the influx of recent thymic emigrants (RTE) Treg into the spleen of the various mutant mice. In *Rag2-Gfp* transgenic mice, these cells can be identified by their remaining low but detectable levels of GFP (**Figure S1F**). We found substantially less Foxp3^+^ Treg among CD4^+^ RTE in spleens of *Il2°*, *Il15°*, and *Il2°Il15°* mice than in wt controls (**Figure 1D**). Taken together, these data indicate that IL-2 and IL-15 non-redundantly and to similar extents control the thymic production of Treg that egress into the periphery.

These data suggest that IL-2 and IL-15 play quantitatively similar roles in Treg development in the thymus. However, we previously showed that Treg recirculating from the periphery inhibit *de novo* development of Treg (28). Since IL-2 plays a crucial role in the survival of Treg in the periphery (29), it would be expected that in *Il2°* mice much less recirculating Treg recirculate to the thymus. We indeed found much less recirculating Treg in thymi of *Il2°* and of *Il2°Il15°* (but not of *Il15°*) mice as compared to wt animals (reduction of 87 ± 5% and 90 ± 6%, respectively, **Figures 1E**, **S1G**). To assess the implication of IL-2 and IL-15 in Treg-development independently of their effect on recirculation of peripheral Treg to the thymus, we quantified Treg in four-day-old mice, in which the thymus only just started to produce Treg and inhibition of Treg development by recirculating cells is minimal (28). Given the consistent results we obtained when analysing Treg-proportions among GFP^+^CD4SP and among GFP^+^ S1P1^+^ CD4^+^CD8^−^ thymocytes in three-week-old mice (*cf*. **Figures 1B, C**), and the observation that Treg only start to leave the thymus at four days of age and are therefore quite rare in the spleen (64), we limited these analyses to GFP^+^ S1P1^+^ CD4SP thymus egress-competent cells. We found substantially (76 ± 4%) less Treg among GFP^+^ S1P1^+^ CD4SP thymocytes in *Il2°* as compared to wt mice (**Figure 1F**). Absence of IL-15 led to a more modest (19 ± 10%) reduction in Treg-development. Analysis of absolute numbers of S1P1^+^ Treg newly developed in four-day-old mice confirmed the major role for IL-2 in Treg-development (**Figure S1H**).

Taken together, our data unequivocally demonstrate that IL-15 and, more prominently, IL-2 play substantial and non-redundant quantitative roles in intrathymic Treg development, which directly impacts the influx of newly developed Treg into peripheral secondary lymphoid organs.

### IL-2 and IL-15 differentially drive development of thymus-exit-competent and periphery-seeding CD25^+^ and CD25^−^ Treg subsets.

The peripheral Treg pool consists of cells expressing or not the IL-2Rα-chain CD25, and CD25^+^ vs. CD25^−^ Treg have distinct properties (65). The observation that IL-2 and IL-15 quantitatively controlled intrathymic Treg development in a non-redundant manner led us therefore to assess expression of CD25 by newly developed Treg.

**Figure 2:**
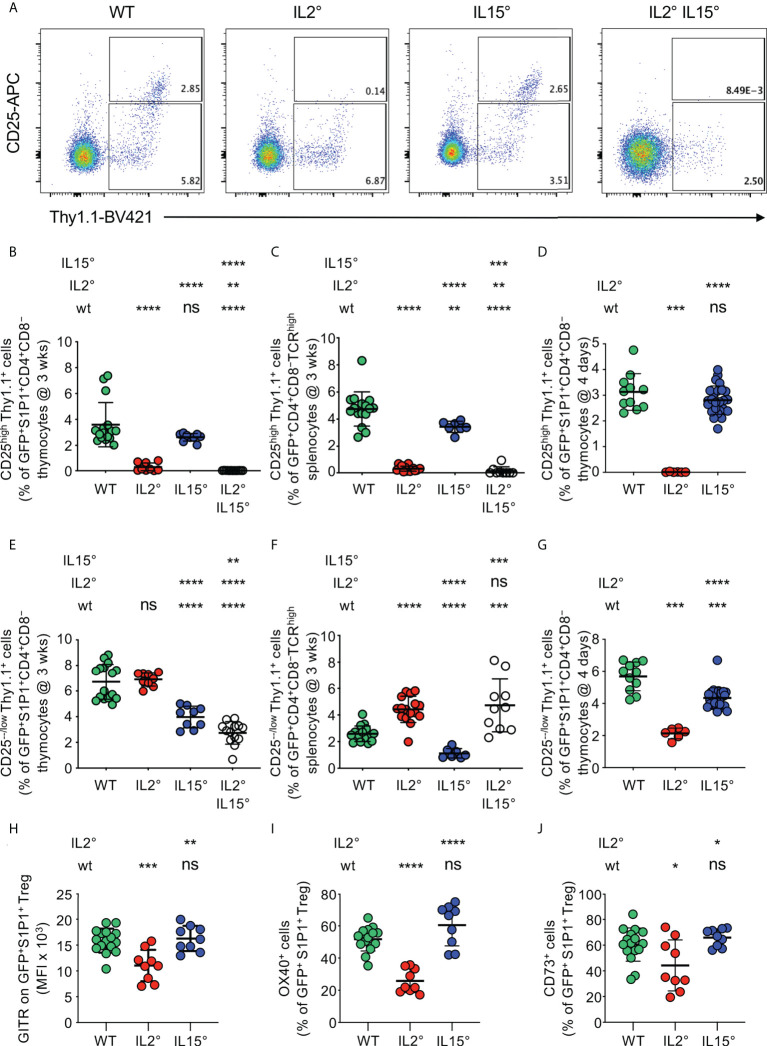
*IL-2 and IL-15 differentially affect the phenotype of thymus exit-competent Treg.* Thymocytes from *Rag-Gfp Foxp3-Thy1^a^* mutant, *Il2°* and/or *Il15°* mice, and *Il2^wt/wt^* and *Il15^wt/wt^* littermates, were stained with fluorescent antibodies against the indicated markers and analysed by flow cytometry. **(A)** Foxp3 vs. CD25 expression by GFP^+^ S1P1^+^ CD4^+^CD8^−^ thymocytes from indicated mice. Depicted gates were used to quantify CD25^−/low^ and CD25^high^ Treg. Percentages of **(B–D)** CD25^high^ or **(E–G)** CD25^−/low^ Treg among **(B, E)** newly developed S1P1^+^ CD4^+^CD8^−^ thymocytes (n=16, 9, 9, and 13 wt, *Il2°, Il15°*, and *Il2°Il15°* mice, respectively) and **(C, F)** CD4^+^CD8^−^TCR^high^ RTE in the spleen from three-week-old animals (n=16, 16, 7, and 9 mice), and **(D, G)** newly developed S1P1^+^ CD4^+^CD8^−^ thymocytes from four-day-old animals (n=11, 6, and 25 mice). **(H–J)** Expression (MFI, % positive cells, as indicated) of indicated markers on GFP^+^S1P1^+^CD69^low^ CD4^+^CD8^−^ thymic Treg from indicated mice (n=16, 9, and 9 mice). Typical cytometry-plots are shown in **Figure S3**. ns, not significant; *p < 0.05; **p < 0.01; ***p < 0.001; ****p < 0.0001 (Mann-Whitney test). Dots indicate individual mice. Bars indicate mean values ± SD.

In *Il2°* and *Il2°Il15°* mice, we found an almost complete lack of newly developed CD25^high^ Treg, while a deficiency in IL-15 did not have a statistically significant impact on the development of this population (**Figure S2A**). Consistently, we found strongly reduced proportions of CD25^high^ cells among newly developed S1P1-expressing Treg in the thymus of *Il2°* and *Il2°Il15°* mice (reductions of 92 ± 9% and 100 ± 0%, respectively) but unaltered percentages in *Il15°* mice (**Figures 2A, B**). Whereas *Il2°* and *Il2°Il15°* mice also displayed a nearly complete (93 ± 5% and 97 ± 6%, respectively) loss of CD25^high^ RTE Treg in the spleen, in *Il15°* mice, we found a smaller decrease of this population (28 ± 9%, **Figure 2C**). We obtained similar results in thymi of four-day-old mice, indicating that they are due to absence of IL-2 or IL-15 and not to differences in accumulation of Treg that had recirculated back from the periphery (**Figure 2D**).

The total absence of newly-developed CD25^high^ Treg in the thymus of *Il2°* mice suggested that development of CD25^−/low^ Treg might be much less affected by the deficiency in IL-2. Importantly, in wt animals, thymus-exit-competent S1P1^+^CD25^−/low^ Treg were even more abundant than S1P1^+^CD25^high^ Treg (**Figure S2B**) and we obtained similar results for RTE in the spleen (**Figure S2C**). Since S1P1-expression is sufficient for thymus-egress (63), these observations indicate that Foxp3^+^CD25^−/low^ CD4^+^CD8^−^ thymocytes can leave the thymus and are not, or at least not exclusively, precursors for Foxp3^+^CD25^high^ newly developing thymic Treg. As compared to three-week-old wt animals, newly developed CD25^−/low^ Treg were abundant and even somewhat increased in the thymus of *Il2°* mice (**Figure S2D**). We also observed unaltered proportions of exit-competent (S1P1^+^) CD25^−/low^ Treg among CD4^+^CD8^−^ cells in the thymus and increased percentages among RTE in the spleen (**Figures 2E, F**). The increase in CD25^−/low^ Treg in the thymus (**Figure S2D**) and among RTE (**Figure 2F**) in *Il2°* mice may be due to (an expected) failure in CD25-expression and/or absence of recirculating Treg. To study the implication of recirculating Treg, we analysed four-day-old *Il2°* mice. As compared to wt animals, we observed strongly (63 ± 6%) reduced levels of newly developed CD25^−/low^ Treg in the thymus of *Il2°* mice (**Figure 2G**). In absence of IL-15, we consistently found substantial reductions in the proportions of CD25^−/low^ Treg among S1P1^+^ Treg in the thymus (reduction of 41 ± 13%) and among RTE in the spleen (reduction of 57 ± 14%) of three-week-old mice, as well as in the thymi of four-day-old animals (reduction of 24 ± 11%, **Figures 2E, F, G**, **S2D**). In thymi of *Il2°Il15°* mice, we found even more reduced proportions of CD25^−/low^ Treg among exit-competent S1P1^+^ CD4^+^CD8^−^ cells than in *Il15°* mice (54 ± 8% vs. 41 ± 13%), indicating non-redundant roles of IL-2 and IL-15 in the development of these cells (**Figure 2E**).

Combined, these data indicate that IL-2 is strictly required for the development of thymus-exit-competent CD25^high^ Treg and, to a lesser extent, also drives that of CD25^−/low^ Treg. By contrast, IL-15 modestly drives development of CD25^high^ as well as of CD25^−/low^ Treg. Since peripheral CD25^−^ vs. CD25^+^ Treg have distinct *in vivo* functional properties (65)(*cf*. discussion-section), these data suggest that IL-2 and IL-15 drive development of functionally distinct Treg.

### IL-2 but not IL-15 modulates expression of GITR, OX40 and CD73 by the newly developed Treg-population

We then searched for further phenotypic differences of Treg developing in wt, *Il2°* and *Il15°* mice. It was previously reported that GITR is expressed at distinct levels on newly developed Treg-subsets (66) and, among several markers studied, we found that OX40 and CD73 are expressed in a bi-modal manner in wt animals (**Figure S3A**). Moreover, in wt animals, GITR, OX40 and CD73 are expressed at higher levels on CD25^high^ than on CD25^-/low^ Treg (**Figures S3A, B**). As a consequence of reduced development of CD25^high^ Treg in *Il2°* mice, average expression levels of these three markers were lower on Treg from *Il2*° than from wt and *Il15°* animals (**Figures 2H, I, J**, **S3A**). Expression of these cell-surface molecules by Treg developing in *Il15*° mice was similar to that on wt Treg (**Figures 2H, I, J**, **S3A**). Together with the data on CD25, these results indicate that IL-2 (but not IL-15) modulates the phenotype of the Treg population newly developing in the thymus and suggest that it may thus affect the functional potential of these cells.

### The TCR-repertoires expressed by Treg that developed in an IL-2 vs. IL-15-dependent manner are distinct

**Figure 3:**
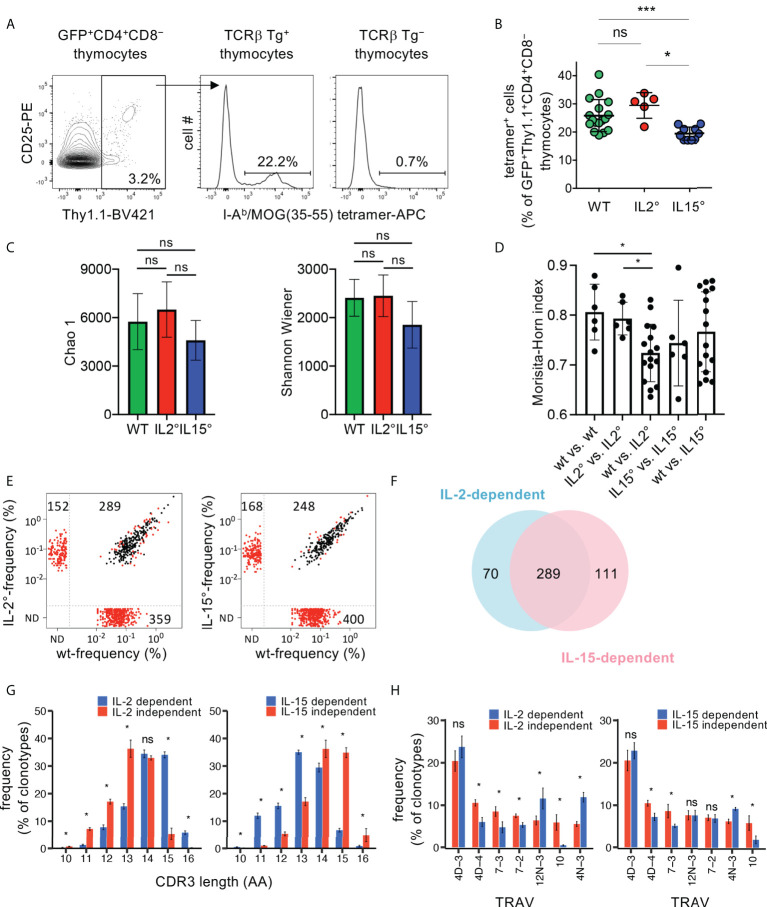
The TCR-repertoires expressed by Treg developing in an IL-2 and IL-15-dependent manner are partially distinct. **(A)** Flow-cytometry analysis of thymocytes from Rag2-Gfp Foxp3-Thy1^a^ Kaa TCRβ-transgenic (Tg^+^) or non-transgenic (Tg^−^) mice. Representative I-A^b^/MOG (35–55) tetramer-stainings on GFP^+^Thy1.1^+^CD4^+^CD8^−^ wt thymocytes (i.e. newly developed Treg) are shown. **(B)** Proportions of tetramer positive cells, gated as in **(A)**, in wt (n=17), Il2° (n=5), and Il15° (n=10) mice. Dots indicate individual mice. **(C–H)** TCRseq analysis of TCRα repertoires expressed by newly developed Treg from Rag2-Gfp Foxp3-Thy1^a^ Tcra^+/^° Kaa TCRβ-transgenic wt (n=4), Il2° (n=4) and Il15° (n=4) mice. **(C)** Chao1 and Shannon-Wiener diversity of the TCR-repertoires. **(D)** Morisita-Horn similarity between all individual samples from indicated mice based on clonotypes with ≥ 5 UMIs. Dots indicate distinct comparisons. **(E)** Frequency of individual clonotypes in the public TCR-repertoire expressed by Treg from indicated mice. Red dots indicate clonotypes differentially expressed between wt and mutant Treg (p< 0.05, LIMMA test). N.D., not detected. **(F)** Venn diagram showing the partial overlap of the IL-2 and the IL-15-dependent public clonotypes (i.e. those within the lower-right quadrants in **(E)**. **(G)** Distribution of the TCRα CDR3-lengths of IL-2 and the IL-15-dependent and -independent public clonotypes (lower-right vs. upper-right quadrants in E, respectively). CDR3α start with conserved Cys and Ala and end with conserved Phe. See **Figure S4** for average CDR3-lengths. **(H)** TRAV- (TCR Vα-segment-) usage in the indicated groups of TCRα clonotypes. Only TRAV represented at ≥5% in at least one indicated group are shown. See **Figures S5**, **S6** for clonotypes found only in cytokine-deficient mice (upper-left quadrants in **(E)**. ns, not significant; *p < 0.05; ***p < 0.001 (Mann-Whitney test). Bars indicate mean values ± SD.

Very localized activity of the IL-2 vs. IL-15 produced by in part distinct thymic (stromal) cell-populations appears involved in Treg development (19, 31). Different thymic stromal cells present distinct self-peptides and thus select Treg with different specificities (33). We therefore hypothesized that the TCR-repertoires expressed by Treg that developed in an IL-2 vs. IL-15-dependent manner are dissimilar. To address this possibility, we bred *Rag2-Gfp Foxp3-Thy1^a^* mice expressing a transgene encoding the public TCRβ clonotype (“Kaa”) of the MOG-reactive CD4^+^ T cell response during EAE in C57BL/6 mice (7, 67), and that were either *Il2°*, *Il15°*, or wt littermates. We analysed the development of MOG (35–55)/I-A^b^-specific Treg by flow-cytometry. A quite substantial proportion (26 ± 6%) of newly developed Treg stained positive with the MOG (35–55)/I-A^b^-tetramer in Kaa TCRβ-transgenic mice, but not in non-transgenic littermates used as controls (**Figures 3A, B**). We did not observe a significant difference between wt and *Il2°* mice. By contrast, we found 22 ± 8% less MOG (35–55)/I-A^b^-specific cells among newly developed Treg in *Il15°* than in wt mice. We conclude that IL-15 plays a significant quantitative role in the development of MOG (35–55)/I-A^b^ -specific Treg while IL-2 appears not involved. These data therefore show different contributions of IL-2 and IL-15 to the development of Treg specific for a self-antigen.

To investigate the involvement of IL-2 and IL-15 in shaping of the TCR-repertoire expressed by Treg in a broader manner, we next bred *Rag2-Gfp Foxp3-Thy1^a^* mice that expressed the Kaa transgenic TCRβ chain, that were heterozygous for a *Tcra* null-mutation, and that were either *Il2°*, *Il15°*, or homozygous wt littermates. We analysed the TCRα-repertoires expressed by newly developed Treg in the thymus by high-throughput sequencing of *Tcra* mRNAs. The diversity (*i.e.* the number of clonotypes and their abundance) of the TCRα-repertoires expressed by Treg populations developing in wt and mutant animals appeared similar (**Figure 3C**). Comparison of the TCR-repertoires demonstrated higher similarities within the four wt and the four *Il2°* replicates than between the four wt and the four *Il2°* samples (**Figure 3D**). We did not find significant differences in the similarities between TCR-repertoires expressed by Treg from wt and *Il15°* mice (**Figure 3D**). These results demonstrate that IL-2 modulates the TCR-repertoire expressed by newly developing Treg. We argued that the principal differences in the TCR-repertoires are the ones reproducibly found in all mice of the same genotype, *i.e*., the “public” TCR-repertoires. *Il2°* mice lacked a substantial part (55%) of the public TCR clonotypes we detected in wt animals (**Figure 3E**). Wt animals lacked 34% of the public TCRs we detected in *Il2°* mice. Similarly, *Il15°* mice lacked 62% of the public TCRs detected in wt animals and wt animals lacked 40% of the public TCRs we detected in *Il15°* mice. A quite large proportion (61%) of the public clonotypes lacking in *Il2°* or *Il15°* mice were identical, and therefore appeared to require both IL-2 and IL-15 for their development (**Figure 3F**). However, 19% of the IL-2-dependent and 28% of the IL-15-dependent public clonotypes appeared to specifically require these respective cytokines. Given that these data concern TCR-clonotypes reproducibly found in wt vs. *Il2° *vs. *Il15°* mice, they strongly suggest that IL-2 and IL-15 contribute to shaping of the TCR-repertoire expressed by newly developing Treg.

To obtain insight into the potentially different characteristics of the public TCRα-clonotypes expressed by Treg developing in an IL-2- vs. IL-15-dependent manner, we next compared their CDR3-lengths and TCR-Vα (TRAV)-usages. The distributions of the CDR3-lengths were different between IL-2- or IL-15-dependent vs. independent clonotypes (**Figure 3G**). The average CDR3-sizes of the public IL-2-dependent clonotypes were somewhat greater than those of the IL-2-independent ones (**Figure S4**). By contrast, the average CDR3-sizes of the public IL-15-dependent clonotypes were slightly smaller than those of the IL-15-independent ones (**Figure S4**). Also clonotypes found in *Il2°* or *Il15°* but not in wt animals had average CDR3-size and distribution of CDR3-lengths that were significantly different from those that were cytokine-independent (**Figures S5A, B**). Analysis of the TRAV-usage revealed substantial differences between cytokine-dependent vs. -independent clonotypes (**Figure 3H**). Also clonotypes found in *Il2°* or *Il15°* but not in wt animals had TRAV-usages that were significantly different from those that were cytokine-independent (**Figure S6**). Taken together, these observations indicate distinct characteristics of the public TCRα-chain clonotypes expressed by Treg requiring the presence or absence of IL-2 or IL-15 for their development. The reproducibility of these results in the four biological replicates for each genotype also indicated that the differences were not due to sampling randomness.

### Origins of the IL-2 and IL-15 involved in Treg development

Probably through trans-presentation by their respective high affinity receptor α-chains, IL-2 and IL-15 can have very local effects (19, 31, 35, 68). Distinct thymic stromal cell-types have distinct phenotypes and thus apparently convey distinct signals to developing T cells (36). Our observation that IL-2 and IL-15 appear to qualitatively modulate Treg development may therefore be due to interactions of developing Treg with distinct stromal cell-types. We therefore sought to identify the stromal cells producing the IL-2 and IL-15 involved in Treg-development, which remains a controversial issue (19, 30–34).

**Figure 4:**
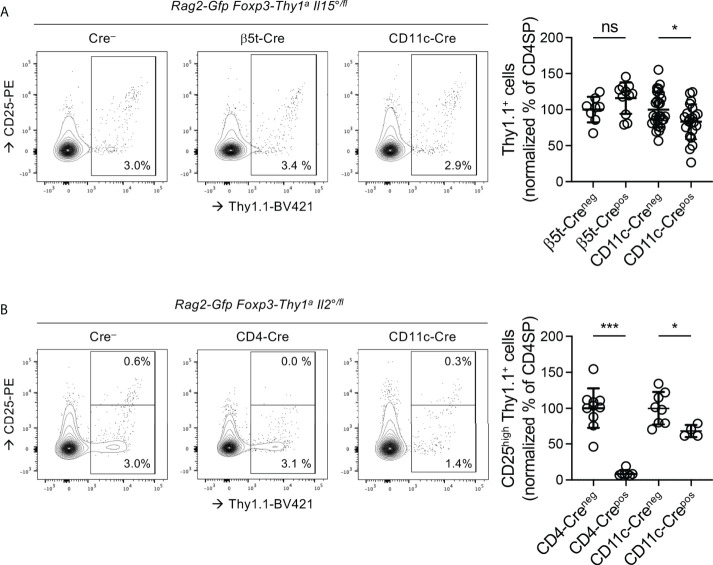
The IL15 involved in Treg development is produced by DC and the IL-2 by T cells and, to a lesser extent, by DC. Thymocytes from three-week-old Rag-Gfp Foxp3-Thy1^a^ mutant, **(A)** Il15°^/fl^ or **(B)** Il2°^/fl^ mice, expressing or not the indicated Cre-transgenes, were stained with fluorescent antibodies against indicated markers and analysed by flow cytometry. (left hand panels) Representative histograms of Thy1.1 (indicating Foxp3-expression) vs. CD25 expression on GFP^+^ (i.e. newly developed) CD4SP thymocytes (gated as in **Figure S1B**). Indicated gates were used for the quantification of (CD25^−/low^ and CD25^high^) Thy1.1^+^ Treg among CD4SP cells. (right-hand panels) Quantification of **(A)** total or **(B)** CD25^high^ Treg among GFP^+^ CD4SP thymocytes. Dots indicate individual mice and values were normalized to the average percentages found in Foxp3^Thy1a/Y^ male and Foxp3^Thy1a/wt^ female, Cre^−^ littermates (**(A)** n=8 β5t-Cre^−^, 11 β5t-Cre^+^, 27 CD11c-Cre^−^, 22 CD11c-Cre^+^, **(B)** 10 CD4-Cre^−^,7 CD4-Cre^+^, 8CD11c-Cre^−^, and 4 CD11c-Cre^+^ mice). ns, not significant; *p < 0.05; ***p < 0.001 (Mann-Whitney test). Dots indicate individual mice. Bars indicate mean values ± SD.

In the thymus, TEC and DC appear to produce IL-15 and to express the IL-15Rα chain required for its trans-presentation to responder cells (19, 32, 34). To assess the role of the IL-15 produced by these cells in Treg-development, we generated *Rag2-Gfp Foxp3-Thy1^a^* mice in which one *Il15* allele was constitutively and the other conditionally invalidated (*Il15°^/fl^*). The Cre recombinase required for invalidation of the *Il15* locus was expressed under control of the promoter of the gene encoding the thymus-proteasome β5t-subunit, active during early stages of TEC-development (47), or that of the gene encoding CD11c, expressed by DC (46). The development of iNKT cells depends on IL-15 (69). *Il15-*invalidation in TEC or in DC led to reduced accumulation of CD4SP iNKT cells (**Figures S7A, B**). Interestingly, *Il15-*invalidation in TEC, but not in DC, led to reduced accumulation of CD4^+^CD8*^−^* iNKT cells (**Figure S7C**). These data thus confirm conditional invalidation of the *Il15* locus in the mice. They also suggest distinct origins of the IL-15 involved in the development of CD4*^−^* CD8^−^vs. CD4^+^CD8*^−^* iNKT cells. Unexpectedly, in β5t-Cre *Il15°^/fl^* mice we found unaltered proportions of Treg among CD4SP thymocytes (**Figure 4A**). By contrast, in CD11c-Cre *Il15°^/fl^* mice (16.8 ± 24.3%) less Treg developed than in control mice not expressing the Cre-recombinase (**Figure 4A**). These results suggest that the IL-15 involved in Treg-development is, at least in part, produced by DC.

Whereas T cells appear to produce IL-2 involved in Treg-development in the thymus, the role of dendritic cells (DC) remains controversial (19, 30, 31, 68). As compared to in their Cre^−^ littermates, in CD4-Cre^+^ *Il2°^/fl^* mice, in which T cells do not produce IL-2, we found very strongly (90.7 ± 4.4%) reduced proportions of CD25^high^ Treg among CD4SP (**Figure 4B**), which confirms that T-cell-derived IL-2 plays an important role in Treg development. Also in CD11c-Cre *Il2°^/fl^* mice, in which DC do not produce IL-2, we found substantially (32.2 ± 8.4%) less newly developed CD25^high^ Treg (**Figure 4B**). These data therefore indicate that IL-2 derived from both T and dendritic cells is involved in Treg-development.

### Thymic Treg from Il2° and from Il15° mice have distinct capacities to prevent autoimmune pathology

**Figure 5:**
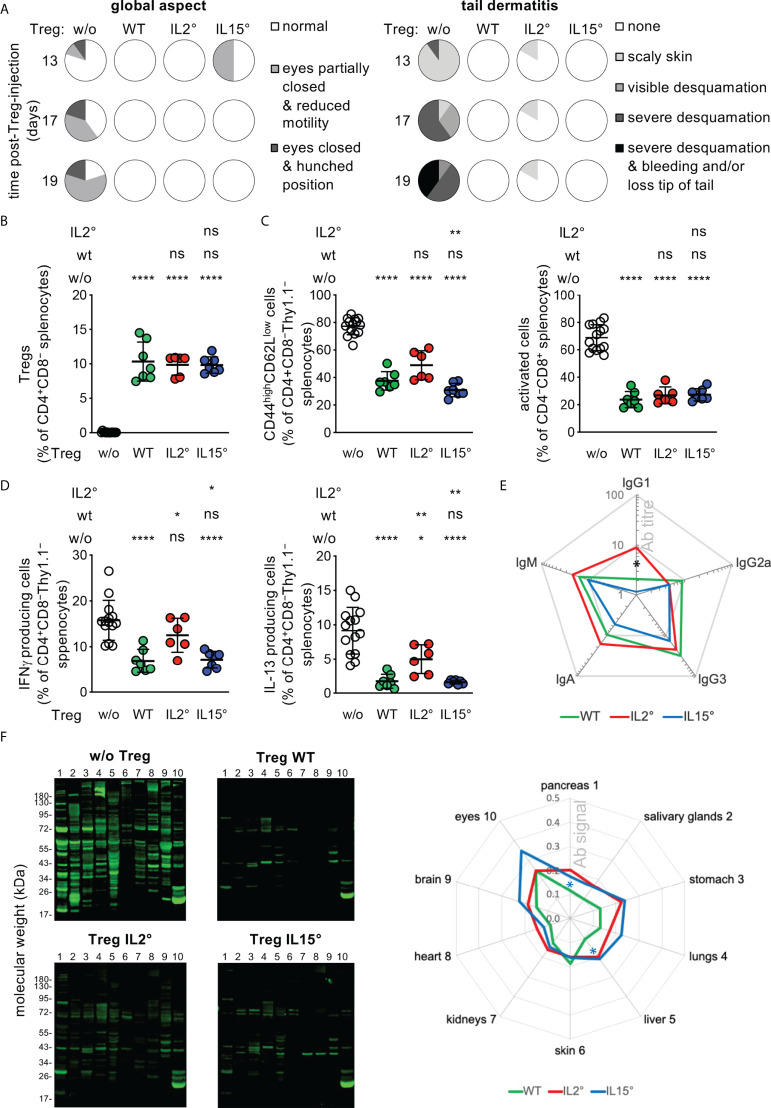
*Treg developing in Il2° vs. Il15° mice have distinct in vivo functions.* Newborn *Foxp3^sf^* mice were i.v. injected with electronically sorted newly developed thymic Treg from three-week-old *Rag2-Gfp Foxp3-Thy1^a^* wt, *Il2°*, or *Il15°* mice, or sham PBS-treated, and analysed three weeks later. **(A)** Eye pathology, reduced motility, hunched posture, and tail-skin-pathology in control *scurfy* mice, and its prevention upon neonatal injection of thymic Treg from wt, *Il2°* and *Il15°* mice. See **Figure S8** for representative pictures of mice, tail close-ups, and lymphadeno- and spleno-megaly. **(B)** Reconstitution of Treg levels in the spleen of *Foxp3^sf^* mice upon injection of wt, *Il2°*, or *Il15°* Treg. **(C)** Proportions of activated (*i.e.* CD44^high^CD62L^low^) CD4 (left) or CD8 (right) Tconv in spleens. **(D)** IFN-γ (left) and IL-13 (right) producing CD4^+^ Tconv in spleens (as determined by flow-cytometry). **(E)** Titres of serum-antibodies of indicated isotypes. “Ab titre” is expressed as mean % of that found in sham-treated *Foxp3^sf^* mice (IgG1, 73.1 ± 35.9; IgG2a, 2.4 ± 2.4; IgG3, 1.2 ± 0.4; IgG2b, 0.0 ± 0.0; IgA, 38.0 ± 6.9; IgM, 137.1 ± 40.6 pg/ml). Mice injected with Treg from wt, *Il2°* or *Il15°* mice had lower titres of all detected antibody-isotypes than sham-treated animals (p ≤ 0.01, Mann-Whitney test). **(F)** Autoantibodies in sera from indicated mice (as determined by incubating Western blots carrying total extracts from indicated *Rag2°* tissues with sera). Representative blots (left panels) and semi-quantification of total signals on indicated tissues (right panel, mean ratios Treg-injected/sham-injected mice) are shown. Numbers on the left side correspond to the tissues indicated on the right side. Statistical significance concern differences between mice that had received IL2°vs. IL15*°* Treg (black asterisk in **(E)** and IL15*°* Treg vs. wt Treg (blue asterisks in **(F)**. Sham-treated *Foxp3^sf^* mice, n=14, 8 independent experiments; *Foxp3^sf^* mice injected with thymic Treg from wt mice, n=7, 3 experiments; from IL-2*°* mice, n=6, 3 experiments; from IL-15*°* mice, n=7, 4 experiments. ns, not significant; *p < 0.05; **p < 0.01; ****p < 0.0001 (Mann-Whitney test). Dots indicate individual mice. Bars indicate mean values ± SD.

Our results reveal that Treg developing in the thymi of wt, *Il2°* and *Il15°* mice are phenotypically distinct and that the TCR-repertoires they express are, in part, distinct. We therefore postulated that Treg from wt vs. *Il2°*vs. *Il15°* mice may have distinct capacities to prevent autoimmune pathology. To assess this possibility, we adoptively transferred (by i.v. injection) identical numbers of GFP^+^Thy1.1^+^CD4^+^CD8^−^ thymic Treg, sorted from three-week-old wt, *Il2°* or *Il15°*, *Rag2-Gfp Foxp3-Thy1^a^* mice, into new-born Treg-deficient *Foxp3^sf^* mice and analysed lymphocyte-activation, cytokine production, (auto)antibody production, and development of immunopathology three weeks later (**Figures 5**, **S8**, **S9**).

Injection of newly developed wt thymic Treg into *Foxp3^sf^* mice reconstituted Treg levels to 11 ± 3% and strongly reduced the symptoms of the autoimmune-pathology observed in sham-treated animals: Skin desquamation; hunched posture; spleno- and lymphadeno-megaly (**Figures 5A, B**, **S8**); activation of splenic CD4^+^ or CD8^+^ T cells (as indicated by a CD44^high^CD62L^low^ phenotype, **Figure 5C**); IFN-γ or IL-13 production by splenic CD4^+^ Tconv (**Figure 5D**); circulation of serum antibodies of IgM, IgG1, IgG2a, IgG3, and IgA isotypes (**Figure 5E**); production of circulating antibodies directed against a large array of autoantigens of all organs assessed (**Figure 5F**), and infiltration by mononuclear cells in pancreas, skin, and lungs, and associated bronchial- and ear-thickening (**Figure S9**). Newly developed Treg isolated from *Il2°* and from *Il15°* thymi populated *Foxp3^sf^* mice as efficiently as wt Treg (**Figure 5B**). They also inhibited development of all of the symptoms, but to distinct degrees (**Figures 5A, C–F**, **S8**, **S9**). Thus, despite similar reconstitution, Treg from *Il2°* (but not those from *Il15°)* mice inhibited to a lesser extent than Treg from wt mice IFN-γ and IL-13 production by CD4 Tconv (**Figure 5D**). Treg from *Il2°* mice prevented the production of IgG1 less efficiently than Treg from *Il15°* mice (**Figure 5E**). Quantification of the tissue blots hybridized with sera from *Foxp3^sf^* mice injected with Treg from wt, *Il2°* or *Il15°* thymi, revealed some minor differences, but all three quite efficiently prevented autoantibody production (**Figure 5F**). We did not find significant differences between the capacity of wt vs. *Il2°* or *Il15°* Treg to prevent infiltration by mononuclear cells in the pancreas, lungs and ears, or thickening of bronchus-walls or ears (**Figure S9**). Combined, these data indicate that the intrathymic availability of IL-2 is essential for the development of a fully functional Treg population. The role of IL-15 in this process appears more subtle.

## Discussion

In the study reported here, we investigated the quantitative and qualitative roles of IL-2 and IL-15 in the intrathymic generation of Treg. By focusing on newly developed Treg and by analysing young mice in which Treg development is practically uninhibited by recirculating Treg, we showed important quantitative roles of IL-2 and, to a lesser extent, IL-15 in Treg development. IL-2 and IL-15 drive the development of phenotypically distinct, thymus egress-competent and periphery-seeding Treg, and differently modulate the selection of the TCR-repertoire they express. Treg developing in absence of IL-2 (but not of IL-15) had clearly detectable, though limited, defects in the control of immune-responses *in vivo*. Combined, these data consolidate and extend the suspected quantitative roles of IL-2 and IL-15 in Treg-development in the thymus and indicate that these cytokines also play important qualitative roles in this process.

The thymi of mice deficient in the IL-2Rα or β chains, in IL-2 or in IL-15 contain fewer Treg (16, 18–20, 23, 70–72). However, among thymic Treg, the proportion of cells that had recirculated from the periphery back to the thymus is very large in wt animals (28). These data therefore mostly failed to formally demonstrate a role for IL-2 and IL-15 in Treg development in the thymus. In our experimental mouse model, we could unambiguously identify newly developed Treg and found substantially less Treg in IL-2- or IL-15-deficient mice than in wt animals. Using an elegant experimental model in which T cell-development was induced through induction of ZAP70-expression, Marshall et al. showed that antibody-mediated IL-2-blockade reduced *de novo* development of Treg, which is consistent with our results (19). It now therefore appears clearly demonstrated that IL-2 affects Treg development in the thymus.

IL-2 and IL-15 do not only act on Treg-precursors but also on other cell-types (potentially) involved in Treg development in the thymus. Thus, IL-2 plays a crucial role in the peripheral survival of Treg (29). Accordingly, in IL-2-deficient mice we found almost no Treg that had recirculated from the periphery back to the thymus. Therefore, as compared to wt mice, in IL-2-deficient mice at least two parameters affecting Treg development had changed: availability of IL-2 and inhibition of Treg development by recirculating cells. To exclude this additional parameter, we analysed Treg-development four days after birth, *i.e.* when Treg just start to develop and leave the thymus, the proportion of recirculating Treg in the thymus is very low, and inhibition of Treg-development is minimal (28). We observed that in absence of IL-2 substantially fewer Treg developed than in absence of IL-15. Our results thus confirm and substantially extend an earlier report in which *de novo* development of Treg was studied through induction of ZAP70-expression (19). Interestingly, we observed a more robust decrease in Treg development in neonate than in three-week-old IL-2-deficient mice (as compared to wt animals). A hypothesis that may explain this observation is that in three-week-old (but not in neonatal) mice, Treg development is substantially inhibited by recirculating Treg, in part through limitation of the availability of IL-2 (28). Further limiting IL-2-availability through genetic invalidation of the gene encoding it would affect Treg development to a lesser extent in three-week-old mice than in neonates, in which recirculating-Treg-mediated inhibition of Treg-development is minimal. Assessing this hypothesis will require the generation of mice in which recirculating Treg do not accumulate in the thymus.

Also iNKT cells may, through production of IL-4, modulate Treg development (73). As we here confirmed, in absence of IL-15 substantially fewer iNKT cells accumulated in the thymus. It remains therefore unclear if the reduced Treg development we observed in IL-15-deficient mice was due to a direct effect on Treg precursors or on iNKT cells. It will be important to study the effect of IL-15 deficiency on Treg development in iNKT cell-deficient mice.

Deficiency in IL-2 or IL-15 will also affect differentiation and/or maintenance of other cell-types known to modulate T cell-development in the thymus but not addressed in this study. For example, IL-15 drives differentiation of CD8 memory T cells (74), reported to reduce negative selection of autospecific thymocytes through deletion of DC and mTEC (75). Therefore, it will now be important to address the involvement of other cell types potentially involved in the effects of IL-2 or IL-15-deficiency on Treg-development.

Combined, our results thus firmly demonstrate quantitatively substantial and non-redundant (direct and/or indirect) roles for IL-2 and, to a lesser extent, IL-15 in Treg development. We also found that the phenotypes of Treg developing in an IL-2- vs. IL-15-dependent manner are distinct: Whereas IL-2 is strictly required for the differentiation of CD25^+^ Treg, IL-15 only plays a modest role, and both cytokines play a role in the development of CD25^−/low^ Treg. Our results thus support and extend an earlier report on the distinct roles of IL-2 and IL-15 in Treg development in the thymus (19). Importantly, in wt animals, the egress-competent phenotype of the thymic CD25^−/low^ Treg subset and the fact that these cells were abundant among RTE in the spleen indicated that it is not (only) a precursor population for newly developing thymic CD25^+^ Treg. The fact that we readily detected these cells among splenic RTE in IL-2-deficient mice supports our conclusion that they do not need to go through a CD25^+^ phase to leave the thymus. Komatsu and colleagues showed that CD25^−^ Treg have a less stable phenotype than CD25^+^ Treg and can lose Foxp3-expression and suppressive activity and acquire the capacity to produce IL-2, IFN-γ, IL-4, and IL-17 (65). *In vitro*, IL-2 did not stabilize Foxp3-expression of peripheral CD25^−^ Treg, suggesting that availabilities of IL-2 in the thymus and in the periphery play non redundant roles. Our results indicate that this apparently functionally distinct CD25^−/low^ Treg subset may, in part, have a thymic origin. Also the GITR, OX-40 and CD73-phenotypes were differentially affected by absence of IL-2 or Il-15. Together, these observations suggest that IL-2 and IL-15 may drive the development of potentially *in vivo* functionally distinct Treg populations.

We hypothesized that IL-2 and IL-15 may drive development of Treg expressing distinct TCR-repertoires. We found that 73% of the public clonotypes observed in wt animals required IL-2 and/or IL-15 for their development. Much smaller proportions of the public clonotypes observed in wt animals specifically required IL-2 (but not IL-15: 11%) or IL-15 (but not IL-2: 17%) for their development. Since distinct proportions of CD25^−^ vs. CD25^+^ Treg developed in *Il2°* vs. wt and *Il15°* mice, these observations are consistent with a recent report showing that intrathymic CD25^−^ vs. CD25^+^ Treg express distinct TCR-repertoires (73). The non-redundant roles of IL-2 and IL-15 may in part be due to an anti-apoptotic action of IL-2 (22) which would allow selection of Treg expressing TCRs recognizing self-MHC/peptide complexes with higher affinity. The public TCRα-repertoires expressed by Treg developing in an IL-2- or IL-15-dependent manner have different CDR3-lengths and TRAV-usages than those developing in wt mice. Previous work demonstrated shortening of the CDR3α during T cell selection in the thymus (76). Interestingly, whereas IL-2-dependent TCRα-chains have larger CDR3 than IL-2-independent ones, IL-15-dependent TCRα-chains have smaller CDR3 than IL-15-independent ones. Distinct CDR3α-lengths and TRAV usages will probably have an influence on the affinities of the TCRs for peptide/MHC complexes expressed by thymic stromal cells, suggesting an interplay between cytokine-receptors and TCR in driving development of the Treg-subsets. Whatever the precise origin(s) of these distinct characteristics may be, they also indicate that the differences in the TCRα-repertoires we observed are not due to sampling biases.

Intriguingly, some Treg newly developing in the thymus of *Il2°* and of *Il15°* mice expressed public TCR-clonotypes that we did not observe in wt animals. Limiting our analysis to clonotypes found in all four replicates will constrain but not entirely avoid effects of sampling randomness, which may therefore be involved in the detection of clonotypes exclusively in *Il2°* or *Il15°* mice. However, small but statistically significant differences in CDR3 lengths and in TRAV-usage of public TCRα found in IL-2*° but not* in wt mice vs. those found in wt *and* in IL-2*°* mice, indicated that a substantial proportion of Treg-clonotypes found exclusively in *Il2°* or *Il15°* mice is not due to sampling biases. By contrast, they suggested that these cells have distinct peptide/MHC-recognition characteristics. A hypothesis that may explain this observation is, again, that signals through the TCR synergize with signals through the receptors for IL-2 and IL-15 to drive differentiation and selection of Treg-precursors, *i.e.* the cytokines would act as a rheostat.

The differences in the TCR-repertoires expressed by Treg from wt, IL-2- and IL-15-deficient mice may be due to the distinct sources of these cytokines in the thymus. Consistent with this postulate, it was previously shown that Treg specific for a model-antigen expressed by mTEC do not require IL-2 for their development (27). IL-2-deficient DC supported the development of Treg less efficiently than wt cells in an *in vitro* thymus culture system (31). In agreement with this observation, we observed a reduction in newly developed Treg in mice with an IL-2-ablation targeted to DC. DC trans-presenting IL-2 *via* the IL-2Rα-chain (35) may favour differentiation of Treg specific for ligands expressed by these stromal cells, as previously suggested (68). Whereas *Il2*-mRNA was detected in thymic DC (31), using an experimental model in which *Il2-*expression-history could be traced, Hemmers et al. did not find evidence for expression in CD90^−^CD19^−^Ly6G^−^Ly6C^−^ thymic DC (30). Together with our data, these observations suggest that IL-2 expression by thymic DC is limited to a particular subset of these cells (or of other CD11c^+^ cells). It appears rather unlikely that the reduced Treg development we observed in the *Il2°^/fl^* CD11c-Cre mice was due to the previously reported very limited activity of the transgenic construct in T lymphocyte-progenitors (<10%) (46). In mice with an IL-15-ablation targeted to DC, we found fewer newly developed Treg, and DC are known to express the IL-15Rα-chain (32). It remains to be investigated if the IL-2Rα and IL-15Rα chains are expressed by the same or by distinct DC and if these DC present the same repertoires of MHC/peptide complexes. Similarly, mTEC trans-presenting IL-15 *via* the IL-15Rα-chain (32) might favour differentiation of Treg specific for ligands expressed by these stromal cells. It was indeed shown that radioresistant stromal cells play an important role in the trans-presentation of the IL-15 involved in Treg development (19). However, even if the abridged development of iNKT cells indicated reduced IL-15 production in our mice with an IL-15-deficiency targeted to TEC, we have not observed a reduction in Treg development. The precise stromal origin of the IL-15 involved in Treg development will therefore require further work.

A non-mutually exclusive explanation for the differences in TCR-repertoires expressed by Treg that had newly developed in wt, IL-2 or IL-15-deficient mice is related to the indirect effects of these cytokines. Both recirculating Treg and iNKT cells (and potentially other cell-types), the presence of which is controlled by IL-2 and IL-15, respectively, appear to modulate Treg development in the thymus (28, 73), and these cells may also modulate selection of the TCR-repertoire, *e.g*. through affecting thymic stromal cells. This postulate would imply that also the TCR-repertoires of Tconv developing in wt vs. *Il2°* vs.*Il15°* animals may be distinct. Assessing these possibilities will require analysis of TCR-repertoires expressed by Tconv and Treg developing in mice lacking recirculating Treg and iNKT cells.

Treg developing in absence of IL-2 or IL-15 therefore are phenotypically (and potentially functionally) distinct and they express distinct TCR-repertoires. We postulated that these cells may have distinct capacities to prevent (auto)immune pathology *in vivo*. To distinguish between roles of these cytokines in the thymus vs. the periphery, we transferred Treg isolated from wt, IL-2 or IL-15-deficient thymi into *Foxp3^sf^* hosts sufficient for these cytokines. Upon transfer into neonatal *scurfy* mice, newly developed thymic Treg from wt, *Il2°* and *Il15°* mice equally efficiently reconstituted adoptive hosts. However, Treg from *Il2°* mice less efficiently inhibited IFN-γ and IL-13 production by CD4 T cells than wt Treg. Consistent with their reduced capacity to inhibit production of a Th2 cytokine, they also less efficiently inhibited serum accumulation of antibodies of IgG1 isotype. Treg from *Il15°* mice had a slightly lower capacity to inhibit formation of autoantibodies to pancreas and liver. However, we did not observe differences in the capacity of newly developed Treg from wt, *Il2°* and *Il15°* mice to prevent the histological lesions observed in Treg-deficient *Foxp3^sf^* mice. These results indicate that IL-2 (and potentially IL-15) plays a qualitative role in the intrathymic development of Treg, a role that cannot be replaced by exposure to this cytokine in the periphery. Redundancy of Treg with distinct antigen-specificities and/or effector-functions may explain the modest nature of the defects we observed.

The reduced capacity of Treg from IL-2-deficient mice to inhibit cytokine production by T cells *in vivo* may be due to reduced stability of Foxp3-expression by these cells. In absence of IL-2, Treg precursors failed to express high levels of CD25. Komatsu and colleagues showed that CD25^−^ Treg have a less stable phenotype than CD25^+^ Treg: They lose Foxp3-expression and suppressive activity, and even acquire the capacity to produce IL-2, IFN-γ, IL-4, and IL-17 (65). Taken together, these two observations suggest that, upon adoptive transfer into IL-2-sufficient mice, (CD25^−^) thymic Treg from IL-2-deficient animals will have a defect in Foxp3-stability and therefore less efficiently control T cell-activation *in vivo*. Extensive (*e.g.* single cell transcriptomic) analysis of the similarity of the CD25^−/low^ Treg-populations developing in wt vs. *Il2*° mice and assessment of their differentiation upon adoptive transfer into neonatal *Foxp3^sf^* mice will be required to assess this hypothesis. Whatever the precise explanation, IL-2 in the thymus and in the periphery apparently has non redundant effects on Treg.

The data presented here indicate a more complex role for the cytokines IL-2 and IL-15 in the intrathymic differentiation of Treg than what was previously appreciated. Rather than only quantitatively controlling this process, they appear to also qualitatively do so by guiding differentiation of Treg with distinct phenotypes and by shaping the antigen-specificity of Treg emerging from the thymus. This is, in part, potentially due to the distinct thymic cell-types known to produce and to respond to these cytokines. The relative availability of IL-2 and IL-15 in the thymus may change during life. We thus previously described that Treg recirculating from the periphery back to the thymus limit the availability of IL-2 and thus inhibit Treg development (28). It will now be important to assess how such potential changes influence the development of Treg in the thymus and the immunosuppressive activity of these cells in the periphery.

## Data availability statement

The datasets presented in this study can be found in online repositories. The names of the repository/repositories and accession number(s) can be found below: https://www.ncbi.nlm.nih.gov/geo/, GSE153484.

## Ethics statement

The animal study was reviewed and approved by the "Comité d’éthique en matière d’expérimentation animale” UMS006 CEEA-122, authorisation number APAFIS#4151-201602171 0481496.v6.

## Author contributions

Conceptualization: CA, OJ, PR, and JM. Methodology: SC and CD. Formal analysis: AG-A and BH. Investigation: CA, NM, SC, CD, HM, JS. Mouse model: BM. Resources: SF, GH, SZ. Writing – original draft: CA, JM; Writing – review & editing: OJ. Supervision: JM. All authors contributed to the article and approved the submitted version.

## Funding

This work was financially supported by the Fondation pour la Recherche Médicale (to JvM, DEQ20160334920); the IdEx Toulouse (to PR); the Région Midi Pyrénées (to JvM, 15/06/ 12.05); the Agence Nationale pour la Recherche (to PR, ANR-16-CE15-0015-01), and the Fondation ARC pour la Recherche sur le Cancer (to CA, DOC20180507201). Il2fl knock-in mice were generated by the Centre d’Immunophénomique (CIPHE, Marseille, France). CIPHE is supported by the Investissement d’Avenir program PHENOMIN (French National Infrastructure for Mouse Phenogenomics; ANR-10-INBS-07 to BM).

## Acknowledgements

We thank Adrian Liston and Pamela Fink for providing *Rag-Gfp Foxp3-Thy1^a^* mutant mice and the NIH tetramer-facility for MOG (35–55)/I-A^b^ tetramers. We are very grateful to the following persons for excellent technical assistance: Fatima L’Faqihi, Valérie Duplan-Eche, Anne-Laure Iscache, Lidia De la Fuente, Paul Menu of the INFINITy Cytometry platform; Adrien Castinel of the GeT-PlaGE Genotoul; and the personnel of the Inserm US006 ANEXPLO/Creffre animal and experimental histopathology facilities. We thank F. Fiore for supervising the construction of the *Il2^fl^* mice and the members of the ‘Integrative T cell Immunobiology team’ and Sylvie Guerder for discussions and input in the project. JPMvM is grateful to the staff of the Biochemistry Institute of the Lausanne University, Epalinges, Switzerland, for its hospitality. CA, AGA, SC, NM, JS, OJ, and JvM dedicate this work to the memory of their late, much respected and regretted colleague PR.

## Conflict of interest

The authors declare that the research was conducted in the absence of any commercial or financial relationships that could be construed as a potential conflict of interest.

## Publisher’s note

All claims expressed in this article are solely those of the authors and do not necessarily represent those of their affiliated organizations, or those of the publisher, the editors and the reviewers. Any product that may be evaluated in this article, or claim that may be made by its manufacturer, is not guaranteed or endorsed by the publisher.
