# Supplementary material for: IL-2 and IL-15 drive intrathymic development of distinct periphery-seeding CD4+Foxp3+ regulatory T lymphocytes
Source: Front Immunol. 2022 Sep 8;13:965303. doi: 10.3389/fimmu.2022.965303 (PMC9495261; doi:10.3389/fimmu.2022.965303)
Supplement: Supplementary file 1 [file DataSheet_1.pdf]

Supplementary Information

- **Supplementary figures**

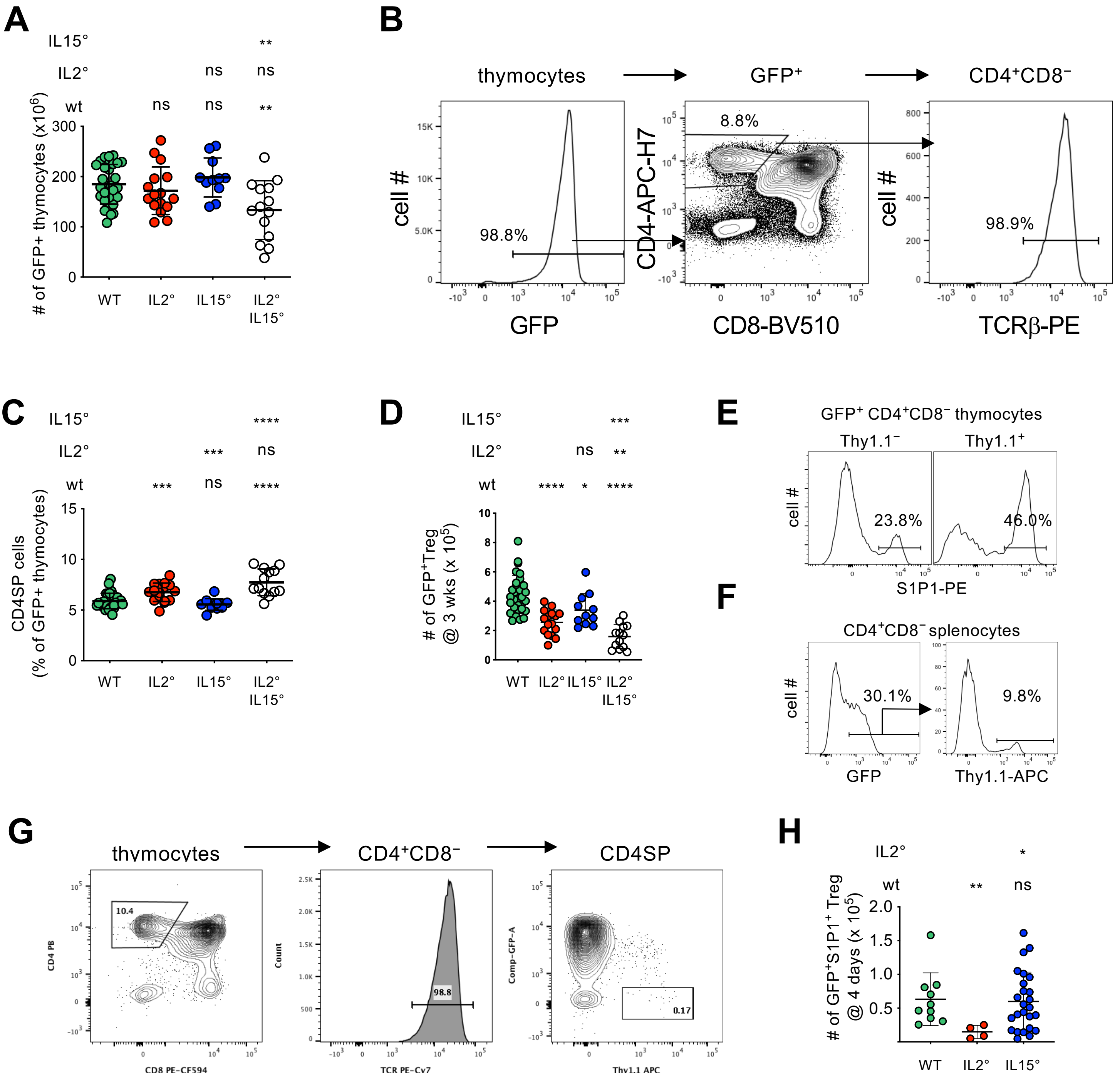

Fig. S1. Treg development in the thymus of wt, IL-2°, IL-15°, and IL-2°IL-15° mice. (**A**) Absolute numbers of GFP$^+$ thymocytes recovered from indicated *Rag2-Gfp Foxp3-Thy1^a* mice. (**B**) Thymocytes from three week-old *Rag2-Gfp Foxp3-Thy1^a* mice were stained with fluorescent antibodies against indicated markers and analyzed by flow cytometry. Subsequent gates allowed electronic selection of live cells (FCS/SSC, not shown), exclusion of doublets (FSC-W/FSC-H, SSC-W/SSC-A, not shown), selection of newly developed (GFP$^+$), CD4$^+$CD8$^-$, and TCRβ^{high} "CD4SP" cells. Thy1.1 expression on thus gated CD4SP thymocytes is shown in Fig. 1A. (**C**) Percentages of CD4SP cells among newly developed thymocytes. (**D**) Absolute numbers of newly developed Treg per thymus. Gating strategy used to (**E**) identify S1P1$^+$ thymic CD4$^+$CD8$^-$ GFP$^+$ Thy1.1$^+$ Treg and Thy1.1$^-$ Tconv and (**F**) quantify Treg among CD4$^+$CD8$^-$ GFP$^+$ cells (RTE) in the spleen. (**G**) Gating strategy used to quantify recirculating (GFP$^-$) Treg among CD4SP thymocytes. (**H**) Absolute numbers of newly developed thymus-egress-competent Treg in four-day-old mice. ns, not significant; *p < 0.05 ; **p < 0.01; ***p < 0.001 ; ****p < 0.0001 (Mann-Whitney test). Dots indicate individual mice. Bars indicate mean values ± SD.

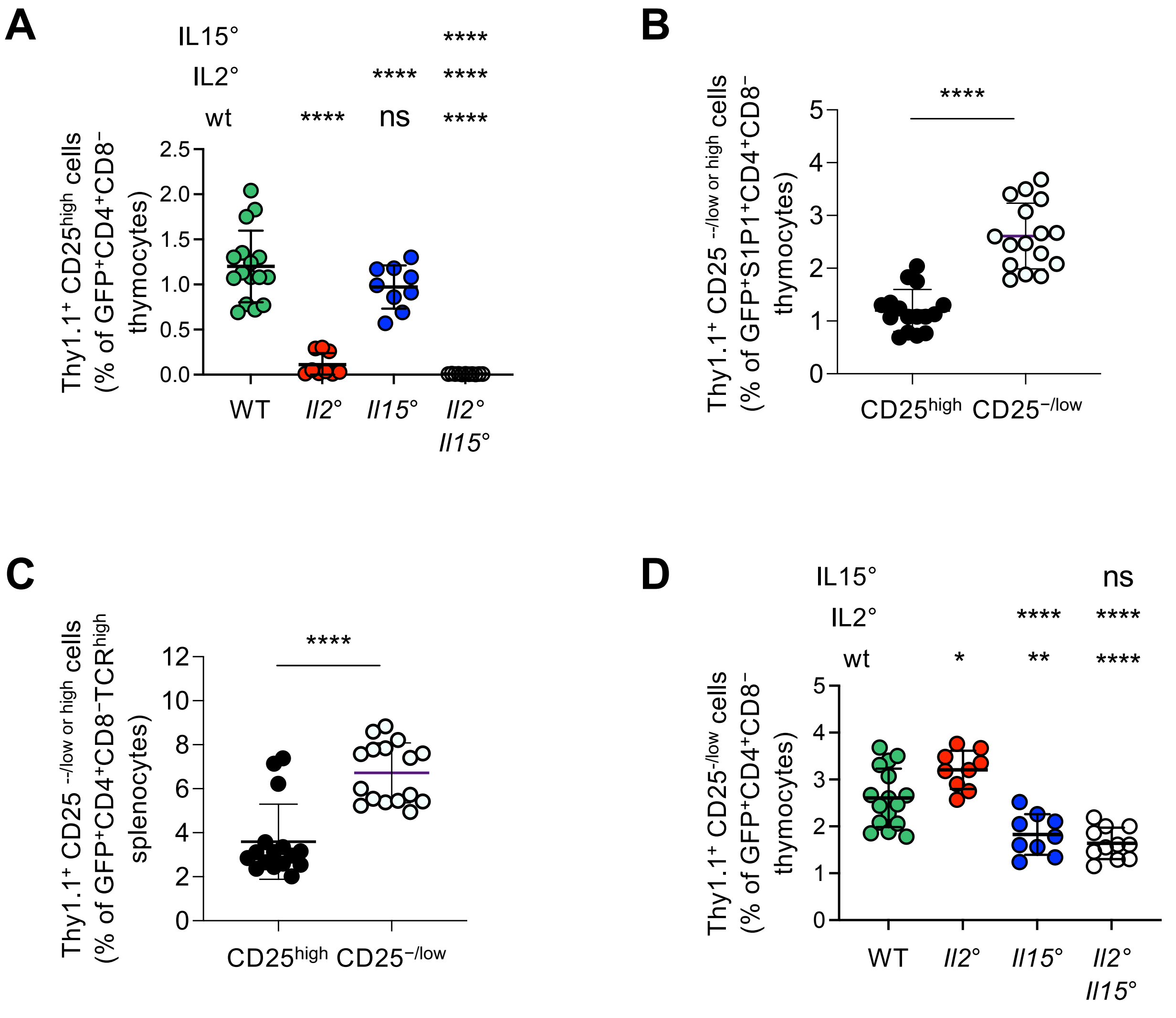

Fig. S2. Expression of CD25 on thymic and splenic T cell populations. Thymocytes or splenocytes (as indicated) from three-week-old *Rag2-Gfp Foxp3-Thy1[a]* (**A, D**) wt, *Il2°*, *Il15°*, and *Il2°Il15°* mice and (**B, C**) wt mice, were stained with fluorescent antibodies to indicated markers and analysed by flow cytometry. (**A**) Percentages of CD25^{high} Treg among newly developed CD4^{+}CD8^{-} cells. Percentages of CD25^{high} and CD25$^{-/low}$ Treg among (**B**) newly developed thymus-exit-competent CD4^{+}CD8^{-} thymocytes and (**C**) CD4^{+}CD8^{-}TCR^{high} RTE splenocytes. (**D**) Percentages of CD25$^{-/low}$ Treg among newly developed CD4^{+}CD8^{-} thymocytes. ns, not significant; *$p < 0.05$; **$p < 0.01$; ****$p < 0.0001$ (Mann-Whitney test). Dots indicate individual mice. Bars indicate mean values ± SD.

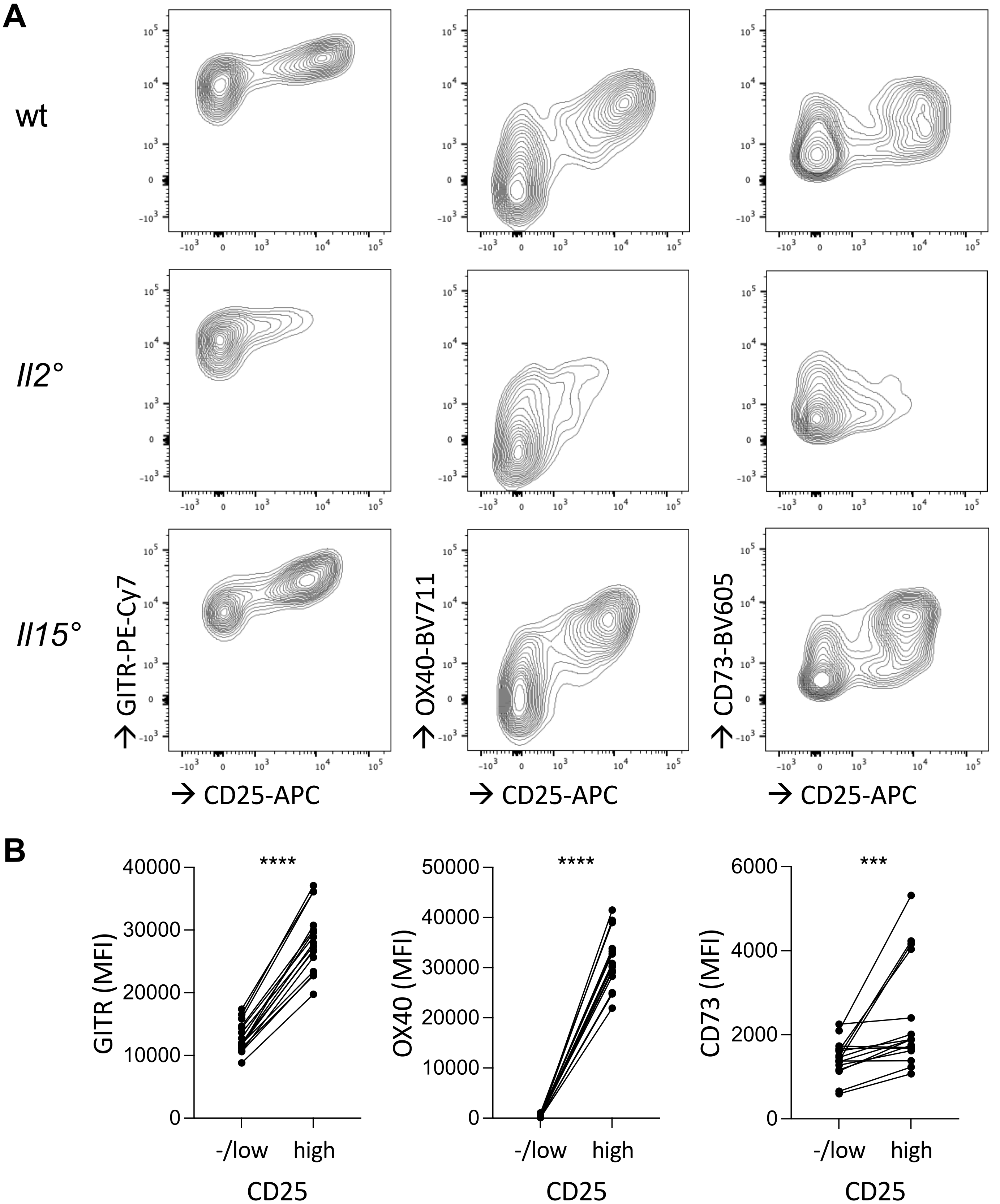

Fig. S3. Expression of CD25 *vs.* GITR, OX40, and CD73 on newly developed Treg in thymi from wt, *Il2°*, and *Il15°* mice. Thymocytes from three-week-old *Rag2-Gfp Foxp3-Thy1ᵃ* wt, *Il2°* and *Il15°* mice, as indicated, were stained with fluorescent antibodies to mentioned and indicated markers and analyzed by flow cytometry. Newly developed and thymus-egress-competent $CD4^+CD8^-$ $Thy1.1^+GFP^+S1P1^+$ Treg were electronically gated and expression of indicated markers depicted. (**A**) Contours plots of indicated markers (depicted are a typical example out of 16 wt, 9 *Il2°*, and 9 *Il15°* animals analyzed). (**B**) MFI of indicated markers on $CD25^{-/low}$ vs. $CD25^{high}$ Treg in wt animals. Data are paired per animal. ***p<0.001, ****p<0.0001, Wilcoxon matched pairs signed rank test.

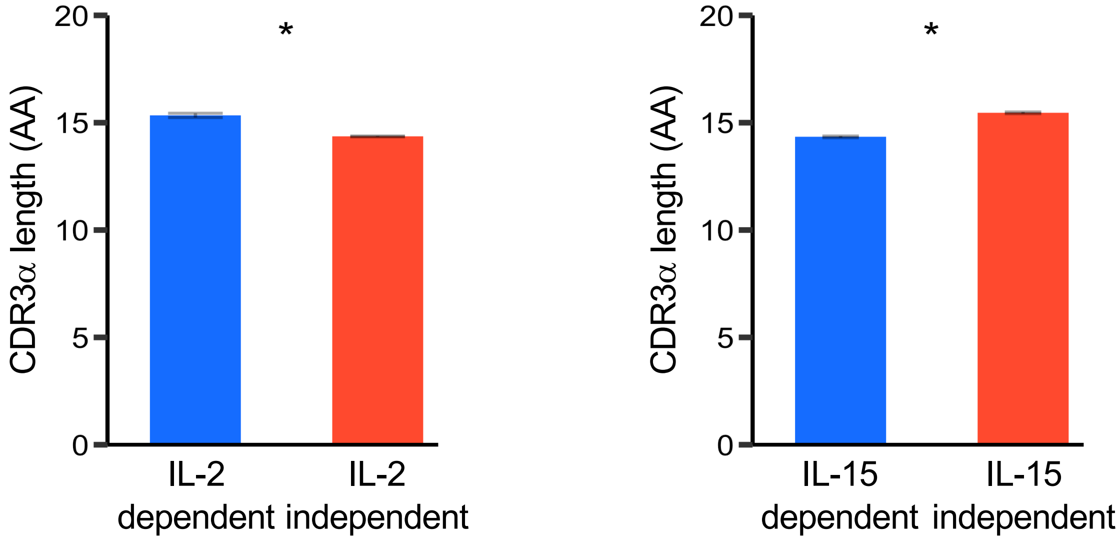

Fig. S4. Average CDR3-lengths of IL-2 or IL-15 dependent *vs*. independent TCRs. TCRseq data obtained as in Fig. 3C-H were processed as described in the Materials and Methods section. TCRα found in all four replicates per genotype were identified for wt and for mutant mice (*i.e*. "public repertoires"). Public TCRα found in Treg from wt but not IL-2° mice are "IL-2 dependent", those found in wt and in IL-2° Treg are "IL-2 independent". *Idem dito* for wt *vs*. IL-15°. CDR3α lengths (expressed in amino acids, AA) of these TCRs in wt animals were determined. Depicted are the average lengths ± SD, n=4, *p<0.05 (Mann-Whitney test).

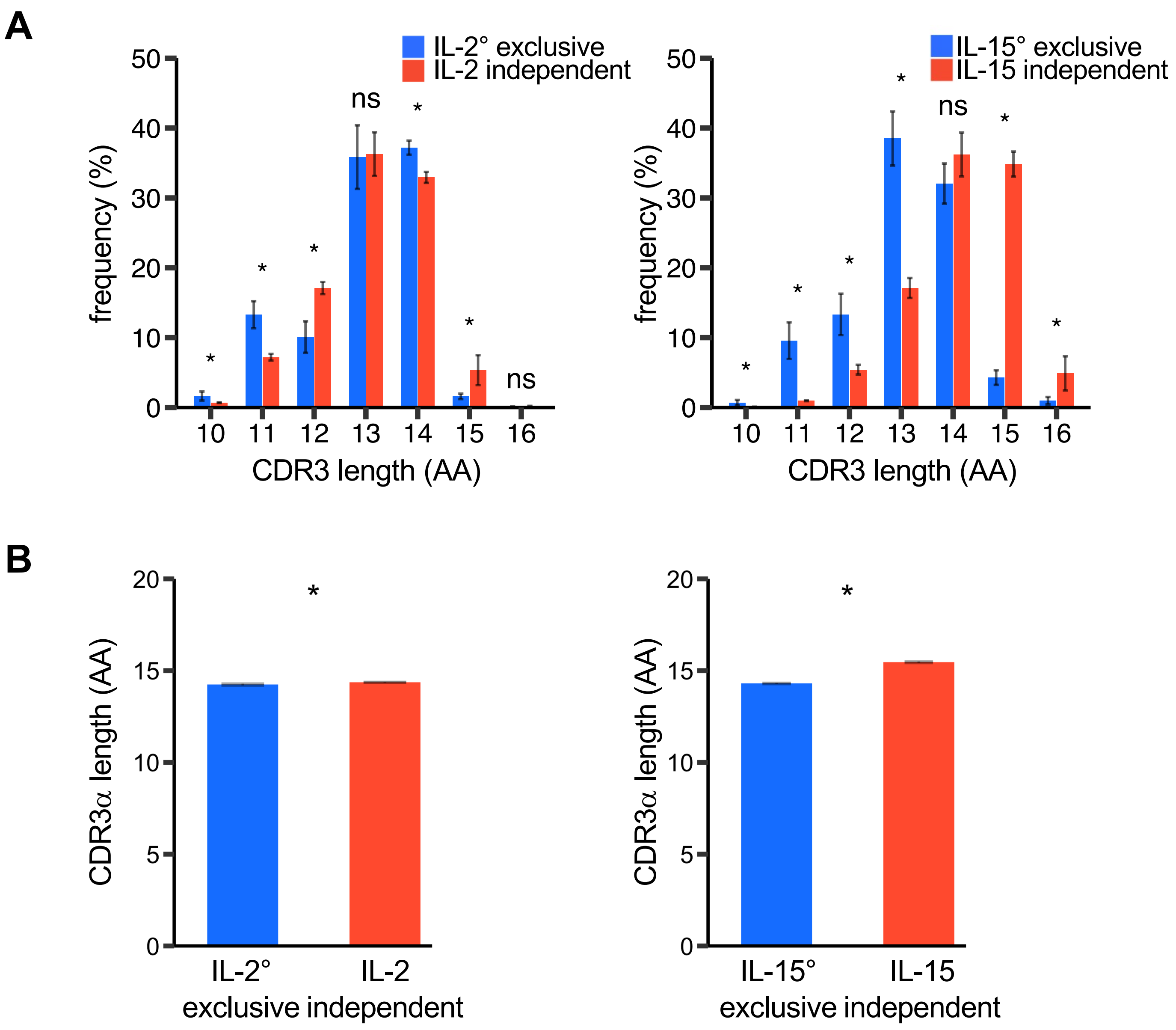

Fig. S5. CDR3-lengths of TCRα found expressed by IL-2° or IL-15° Treg but not by wt Treg. TCRseq data obtained as in Fig. 3C-H were processed as described in the Materials and Methods section. TCRα found in all four replicates per genotype were identified for wt and for mutant mice (*i.e.* "public repertoires"). Public TCRs found in Treg from IL-2° but not wt mice are "IL-2° exclusive", those found in wt and in IL-2° Treg are "IL-2 independent". *Idem dito* for wt *vs.* IL-15°. CDR3α lengths (expressed in amino acids, AA) of these TCRα in IL-2° or IL-15° animals were determined. Depicted are (**A**) the CDR3α-length distribution and (**B**) the average lengths ± SD, n=4. ns, not significant; *p<0.05 (Mann-Whitney test).

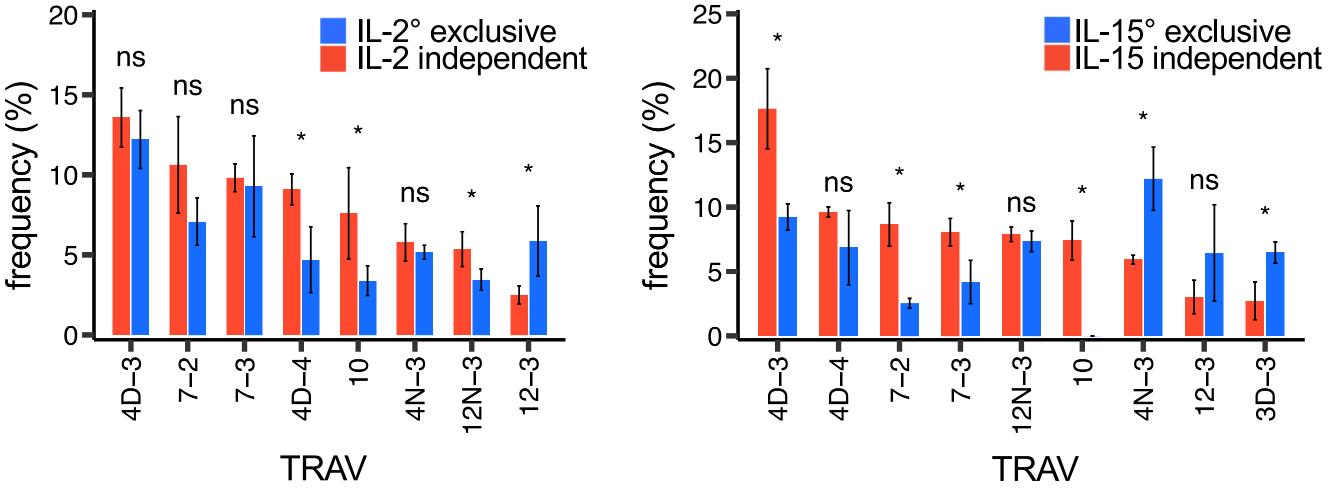

Fig. S6. TRAV-usage by TCRs found expressed by IL-2° or IL-15° Treg but not by wt Treg. TCRseq data obtained as in Fig. 3C-H were processed as described in the Materials and Methods section. TCRα found in all four replicates per genotype were identified for wt and for mutant mice (*i.e.* "public repertoires"). Public TCRs found in Treg from IL-2° but not wt mice are "IL-2° exclusive", those found in wt and in IL-2° Treg are "IL-2 independent". *Idem dito* for wt *vs*. IL-15°. TRAV-usages by these TCRα in IL-2° or IL-15° animals were determined. Depicted is the average representation of TRAVs observed at a frequency of ≥5% in at least one of the two groups ± SD, n=4. ns, not significant; *$p<0.05$ (Mann-Whitney test).

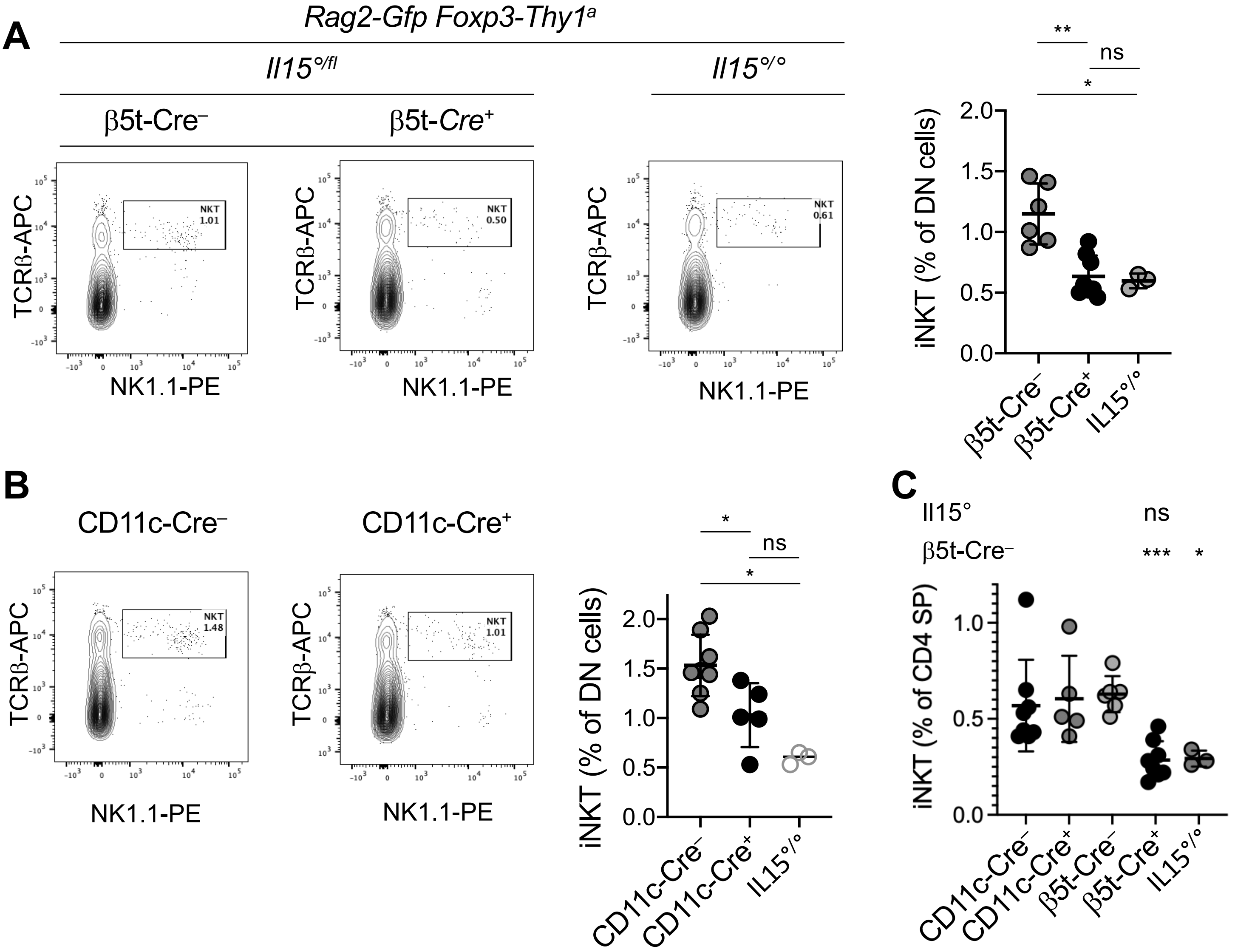

Fig. S7. iNKT cell-development in conditionally IL-15-deficient mice. (**A-C**) Thymocytes from three-week-old *Rag2-Gfp Foxp3-Thy1a Il15°/fl* or *Il15°/°* mice (as indicated) expressing (or not) the indicated Cre-transgenes were stained with fluorescent antibodies to CD4, CD8, TCRβ, and NK1.1 and analyzed by flow cytometry. (**A, B**) Typical NK1.1 *vs*. TCRβ patterns of $CD4^-CD8^-$ thymocytes (left panels) and thus found percentages of iNKT cells (gated as in left hand panels) among $CD4^-$ $CD8^-$ (DN) cells. **(C)** Percentages of iNKT cells among $CD4^+CD8^-TCR\beta^{high}$ (CD4SP) thymocytes. ns, not significant; *p < 0.05; **p < 0.01; ***p < 0.001 (Mann-Whitney test). Dots indicate individual mice. Bars indicate mean values ± SD.

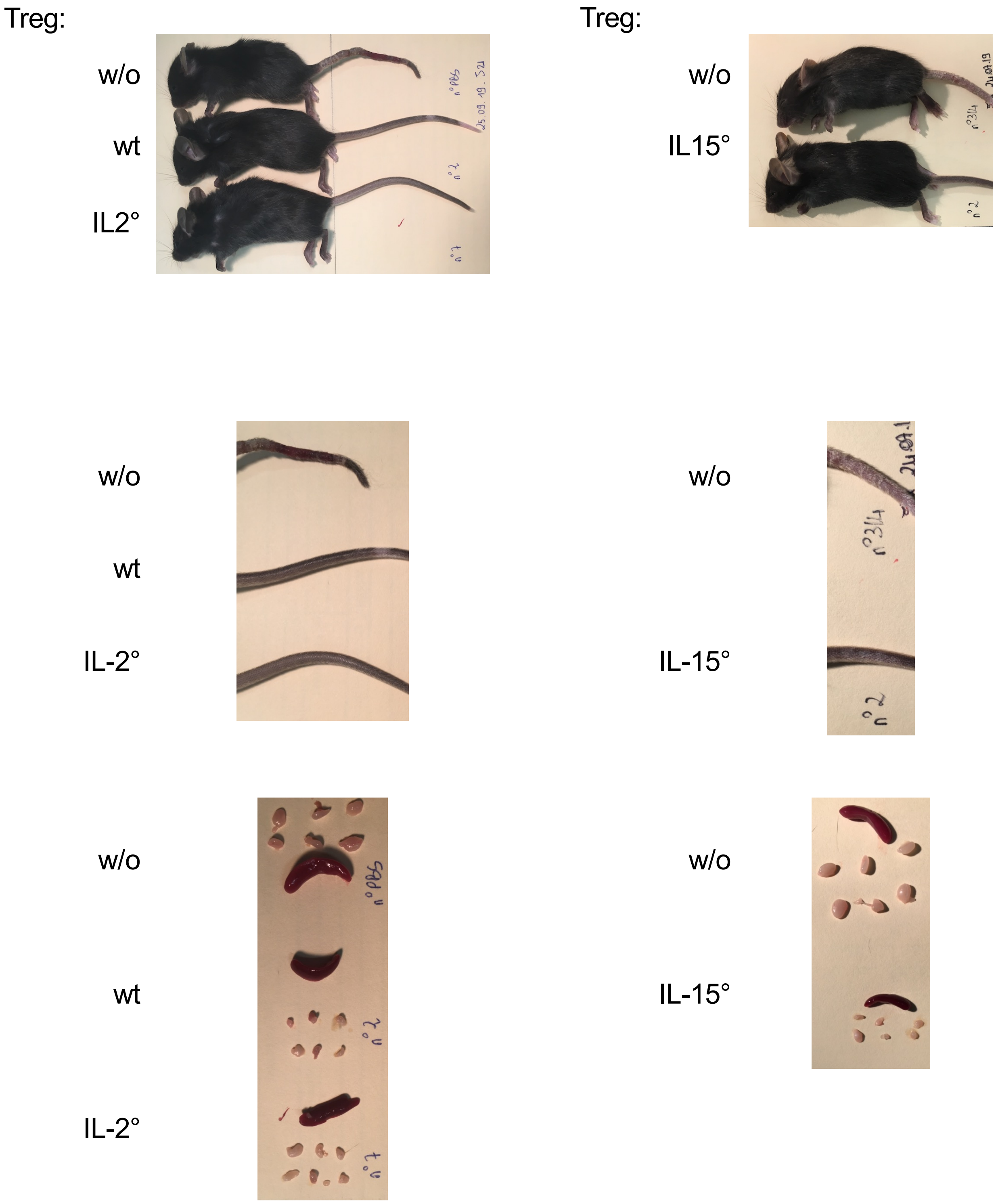

Fig. S8. Macroscopic analysis of tail and lymphoid organs of *Foxp3*^{sf} mice reconstituted with Tregs. *Foxp3*^{sf} mice were i.v. injected at birth with newly developed thymic Tregs sorted from three-week-old wt, *Il2*° or *Il15*° mice (or with PBS as control). At three weeks of age, mice were euthanised and analysed. Photos were taken to compare hunched aspect and reduced growth (top), aspect of the tail (skin desquamation, lesions)(middle) and lymphoid organs (lymphnode-pairs, from the left to the right: axial, brachial and inguinal, spleens)(bottom).

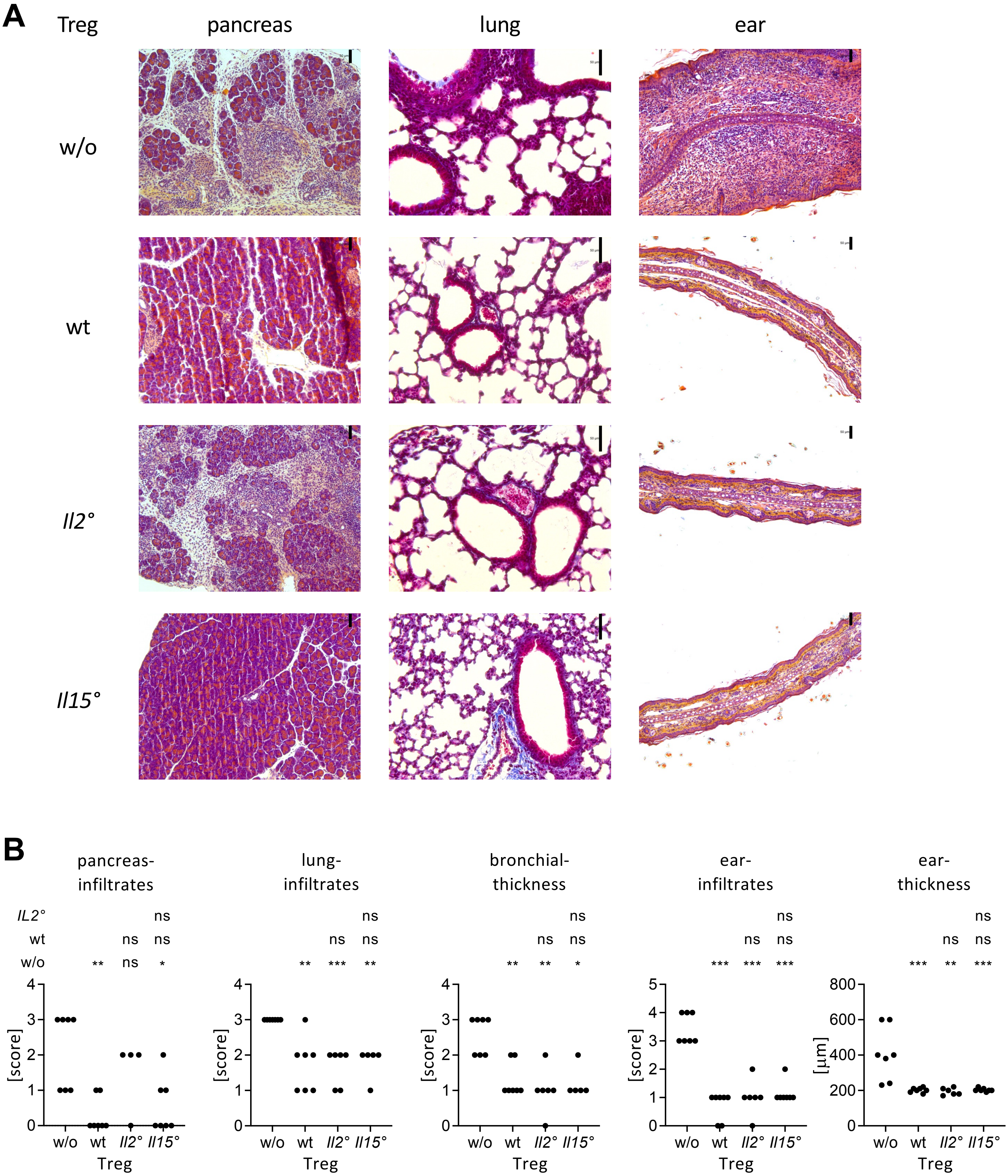

Fig. S9. Histological analysis of organs from *Foxp3*^{sf} mice neonatally reconstituted with newly developed Tregs from wt, *Il2*°, or *Il15*° thymi. (**A**) *Foxp3*^{sf} mice were i.v.-injected at birth with newly developed thymic Tregs from three-week-old wt, *Il2*° or *Il15*° mice (or with PBS as control). At three weeks of age, mice where euthanised, organs removed, tissue sections H/E stained and analysed by microscopy. Size bars, 50 µm. (**B**) Attributed scores of indicated symptoms and thickness of ears. ns, not significant; *p<0.05; **p<0.01; ***p<0.001 (Mann-Whitney test)
